# Factors influencing emotional driving: examining the impact of arousal on the interplay between age, personality, and driving behaviors

**DOI:** 10.3389/fpsyg.2025.1487493

**Published:** 2025-02-10

**Authors:** Zhegong Shangguan, Xiao Han, Younesse El Mrhasli, Nengchao Lyu, Adriana Tapus

**Affiliations:** ^1^Autonomous Systems and Robotics Lab, Unité d'Informatique et d'Ingénierie des Systèmes (U2IS), ENSTA, Institut Polytechnique de Paris, Palaiseau, France; ^2^School of Systems Science, Beijing Jiaotong University, Beijing, China; ^3^Intelligent Transportation Systems Research Center, Wuhan University of Technology, Wuhan, China

**Keywords:** driving style, driving behavior, personality, individual differences, user profile, emotional arousal

## Abstract

**Introduction:**

Drivers' emotions have been widely investigated in transportation due to their significant effects on driving behaviors and traffic accidents. Appraisal theory posits that emotional reactions are influenced by individuals' attitudes toward current circumstances and events, thereby shaping their driving attitudes and styles. However, In the study of emotional driving, research often focuses on the impact of single factors such as age, gender, and personality, while the interplay between these multiple factors is a challenge. This study aims to explore the impact of age, personality, and driving experience on driving behaviors, and to investigate the interaction effect between these factors, particularly the role of emotional arousal.

**Method:**

Using moderated moderation and mediated moderation analyses, we examined how these individual factors interact and influence driving behaviors, including acceleration, speed stability, and steering performance. Data were collected from a driving simulation experiment involving 40 Chinese participants in various emotional states.

**Results:**

Our findings revealed that higher-age drivers and experienced drivers displayed lower maximum acceleration and better speed stability. Extraversion significantly mediated the relationship between age and driving behaviors, with this relationship being moderated by arousal states. Additionally, Neuroticism moderated the relationship between driving experience and driving behaviors.

**Conclusion:**

This study highlights how individual factors influence the trajectory of personality development in relation to driving behaviors. These findings have practical implications for improving traffic safety and driver education programs by incorporating emotional and personality-based interventions. Further long-term and individualized studies are needed to better understand these interactions and develop targeted interventions.

## 1 Introduction

Road traffic accidents continue to pose a significant threat to individuals worldwide, with approximately 1.25 million fatalities occurring each year (Forum, 2020; Zhang et al., 2019). As we moved into the post-pandemic phase of COVID-19, the importance of addressing road safety becomes even more evident. According to the Road Safety Annual Report 2022, there has been a concerning trend of increasing road deaths in several countries, even compared with the pre-pandemic era. On average, road deaths increased by 0.1% in 2021 across the 34 International Road Traffic and Accident Database (IRTAD) countries, compared to the average from 2017–2019 (Forum, 2022). Particularly alarming is the surge in road deaths in the United States, which experienced a 16.3% increase to reach 42,915 fatalities in 2021, compared to the average from 2017 to 2019. These statistics underscore the urgent need for effective measures to enhance road safety and protect road users.

In recent years, numerous policies and technological advancements have been implemented to reduce traffic accidents, with many IRTAD partners achieving positive results cross all road safety indicators. Norway, in particular, has emerged as a success story Its efficient approach to assessing road safety risks for different population groups has led to significant improvements (Iversen et al., 2005; Nordfjærn et al., 2011; Liu et al., 2022). The success of Norway and previous research highlight the importance of studying individual differences to enhance road safety. By understanding factors such as age, personality, driving experience, and behavior, tailored interventions can be developed to address specific risk groups and traffic scenarios. This highlights the significance of invstigating individual differences and their implications for promoting road safety.

Investigating individual differences and user profiles among drivers is crucial addressing the persistent issue of road accidents, as human factors such as age, driving experience, personality, emotions, and distraction are the leading causes of crashes (Zhang et al., 2019; Habibifar and Salmanzadeh, 2022; Masello et al., 2023). Of particular interest is the exploration of emotional driving behaviors, considering that emotion plays a crucial role in perception (Vuilleumier, 2005) and decision-making (Forgas, 1995). Understanding drivers' emotions and their relevance to traffic safety are essential for transportation research (Steinhauser et al., 2018; Kadoya et al., 2021), especially with the growing number of vehicles and changing road conditions such as high-density traffic. Drivers can hardly stay in a stable state free from feeling, and emotional driving states can lead to traffic violations, motivational tendencies, and maladjusted driving behaviors, such as aggressive driving and distraction (Adams et al., 2006; Harmon-Jones, 2003; Roidl et al., 2014).

Emotional regulation and responses can be influenced by two distinct pathways: antecedent-focused and response-focused strategies (Gross, 2013). While personality is typically considered an individual's self-adjustment, personality traits have a profound impact on the response-focused emotional regulation strategy (Purnamaningsih, 2017). Personality traits can act as mediators by influencing how an individual responds emotionally to a given situation. Based on the Big Five personality model (John et al., 1991), research has revealed links between different personality traits and specific emotion regulation strategies. For example, Extraverted individuals tend to experience more positive emotions and may avoid expressing the opposite, while Introverted individuals are more inclined to avoid or withdraw from various situations (Gross, 2013). Additionally, a complex relationship between personality and affect, suggesting that Extraversion and Neuroticism contribute to affect through emotion regulation simultaneously, was reported in Wang et al. (2009).

Factors like gender and age play critical roles, especially under high arousal states. Males tend to down-regulate negative emotions more effectively, possibly due to differences in the autonomic nervous system (Nelis et al., 2011; Dart et al., 2002). Older individuals exhibit more effective emotional regulation and suppression (Brummer et al., 2014). Young drivers, despite being a small percentage of licensed drivers, are responsible for a disproportionate number of fatal accidents (Rahman et al., 2021). Given the high accident rates among young drivers and an aging population (Bergen et al., 2017), investigating age variation and experience in driving behavior is crucial.

According to the theory of appraisal, emotional reactions help individuals prepare for and respond appropriately to potential harm or benefit (Doan et al., 2024; Demutti et al., 2022; Scherer, 1999). Subjective experiences and attitudes toward driving tasks exhibit significant variations among individuals, influenced by factors such as gender, personality, and culture (Kuppens et al., 2013, 2017). Research has identified different driving styles or attitudes toward driving tasks (Taubman-Ben-Ari et al., 2004; Eboli et al., 2017), and individual differences can impact driving behaviors, including speed or steering wheel behaviors. High arousal states can enhance driving performance (Eason et al., 1969; Ünal et al., 2013) and influence the driver's perceptive area (Zhang et al., 2016). Thus, understanding the causal factors and interactions among human factors in emotional driving scenarios is crucial for preventing fatal road traffic collisions (RTCs) in the early stage.

Despite extensive research on driving behavior under different emotions (Trick et al., 2012; Roidl et al., 2014), the underlying factors behind these emotions are often overlooked. Several research gaps and challenges remain to be addressed. Firstly, previous studies have primarily focused on how different emotional states affect driving behavior variations, with less attention given to the influence of stable factors or traits that shape these behaviors, such as acceleration, speed stability, and steering performance. Therefore, further investigation is needed to understand how personality influences emotional responses in driving contexts. Secondly, this gap is exacerbated by the scarcity of culturally specific research, as most studies concentrate on Western populations, leaving regional or cultural differences largely unexplored. The variety of emotional responses across cultures may reveal insights into the nature of emotion. By addressing these gaps, our study aims to provide a more comprehensive understanding of the interplay between emotions, personality traits, and driving behaviors, particularly in the context of Chinese drivers.

This study introduces a novel approach by applying moderated moderation and mediated moderation analyses to explore the complex interconnections between age, personality traits, driving experience, and driving behaviors in Chinese drivers, while accounting for the moderating effects of arousal states. By analyzing empirical data (Li et al., 2022), the findings reveal several significant insights: (1) age positively predicts Extraversion, which mediates the relationship between age and driving behaviors, amplifying risky behaviors under high-arousal conditions; (2) Neuroticism moderates the relationship between driving experience and driving stability, with high Neuroticism diminishing the benefits of driving experience; and (3) arousal states interact with personality traits, particularly Extraversion, to influence driving behaviors, emphasizing the interplay between situational and individual factors.

These results contribute to the literature by offering a deeper understanding of how individual differences and emotional states shape driving behaviors. Specifically, the study provides evidence for cultural factors influencing personality expression in driving, highlights the importance of considering emotional regulation in traffic safety interventions, and underscores the value of tailored approaches to driver training and safety programs based on personality traits and age.

The structure of this paper is as follows: Section 2 provides a comprehensive literature review, including theoretical foundations and the development of hypotheses. Section 3 describes the methodology employed, including sample characteristics and experimental design. Section 4 outlines the analytical techniques and statistical models used to examine the relationships between age, personality traits, driving experience, and driving behaviors. Section 5 presents the empirical findings. Section 6 discusses these results in the context of the existing literature, discussing practical implications, limitations, and future research directions. Finally, Section 7 summarizes the study's key contributions and recommendations.

## 2 Literature review and hypotheses

### 2.1 Valence and arousal

Emotions arise from a combination of internal and external factors, such as personal events, environmental conditions, and social interactions, and they can substantially influence cognitive processes-including attention, decision-making-and subsequent behaviors. Recognizing how these affective states shape actions is particularly vital in high-stakes contexts like driving.

Appraisal has been widely adopted in emotion research, positing that people evaluate or “appraise” events to generate specific emotions (Smith and Lazarus, 1993; Roseman, 1984). Building on this, Russell's Emotion Circumplex model conceptualizes emotional states along two or three core dimensions-valence, arousal, and sometimes dominance (Russell, 1980).

- Valence refers to the positivity or negativity of an emotional experience (ranging from sad or angry to happy or joyous).
- Arousal captures the intensity of physiological and psychological activation (from calm to excited).
- Dominance describes the extent of control or influence an individual feels.

**Figure 1:**
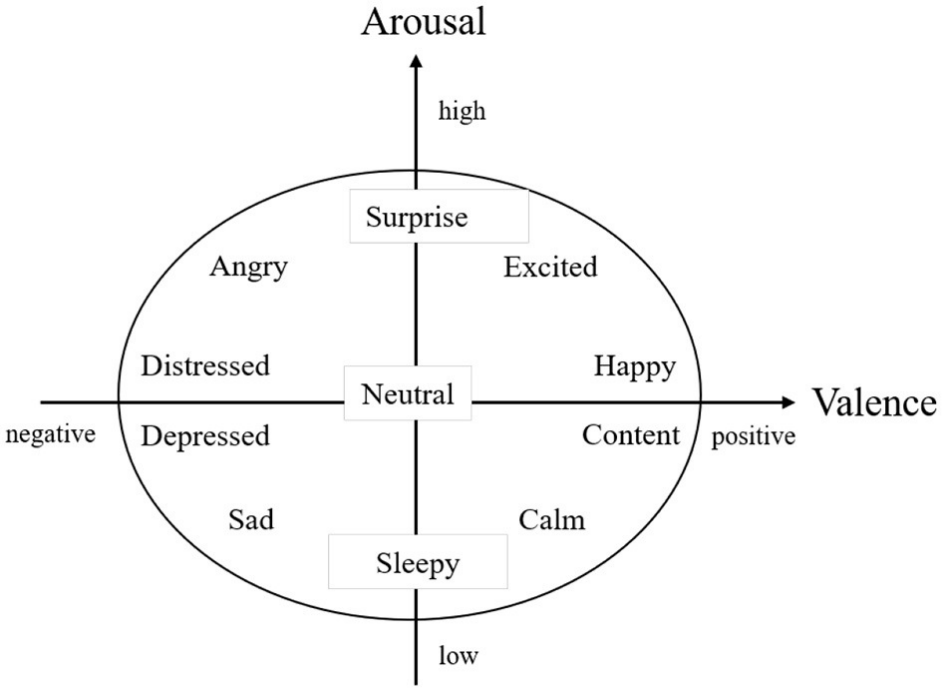
Valence and arousal (Tsiourti et al., 2019).

These dimensions allow a spectrum view of emotion rather than focusing on discrete emotional labels. Accordingly, the Valence-Arousal-Dominance (VAD) framework has been applied to numerous settings, but the Valence-Arousal (VA) model often suffices for understanding essential affective processes (Juvina et al., 2018). Figure 1, adapted from Tsiourti et al. (2019), illustrates the conceptual placement of emotional states along these axes. Measurement approaches often rely on self-reported questionnaires to assess valence and arousal (Habibifar and Salmanzadeh, 2022). Researchers also induce emotions in participants through autobiographical recollection (Steinhauser et al., 2018), video clips (Zhang et al., 2016), or immersive simulations. This methodology has facilitated a substantial body of work examining how emotions, especially those characterized by high arousal, impact drivers' performance.

Numerous studies show that both valence and arousal strongly modulate driving outcomes. For instance, Du et al. (2020) found that positive emotions improved takeover performance in automated vehicles, while Zhang et al. (2022b) demonstrated that high-arousal negative emotions (e.g., anger) can lead to risky maneuvers. Similarly, background music with high-arousal characteristics enhances reaction times (Ünal et al., 2013), whereas heightened physiological arousal-often measured via heart rate (BPM)-can negatively affect social coping factors such as fear of negative evaluation (Sutherland et al., 2022).

Specifically, anger has been repeatedly associated with increased mean speed, greater speed variability, and more frequent errors compared to emotions such as fear (Zhang et al., 2022b; Roidl et al., 2014; Jeon et al., 2011; Abdu et al., 2012). Anxiety can undermine situational awareness, prompting failures to check mirrors or yield to oncoming traffic (Bernstein et al., 2019), while depression may lead to fatigue or lapses in concentration that elevate collision risks. Notably, around 16% of the general population reports moderate to severe driving anxiety-a high-arousal, negative-valence state that heightens concerns about road rage and lowers perceived driving safety (Taylor, 2018). Moreover, spatial anxiety, a domain-specific variation of anxiety, can exacerbate risky driving by leading to over-regulation and more frequent lapses behind the wheel (Traficante et al., 2024).

Collectively, these findings confirm that high-arousal negative emotions frequently result in more dangerous driving (e.g., speeding, erratic lane changes), whereas positive valence or low-arousal states can mitigate these risks (Hu et al., 2013). Building upon this literature, our study moves beyond simply linking emotions to driver performance by examining which stable individual differences-particularly personality traits-may contribute to these emotional responses and driving outcomes.

### 2.2 The role of personality traits

Personality traits have long been recognized as robust predictors of individual differences in both affective responses and behaviors (Taubman-Ben-Ari et al., 2004; Taubman-Ben-Ari and Yehiel, 2012; Kuppens et al., 2017; Steinbakk et al., 2019). In driving research, Eysenck's P-E-N theory (Psychoticism, Extraversion, Neuroticism) has been influential for explaining why some individuals are more susceptible to risky driving under emotional arousal (Eysenck, 1963; Tinella et al., 2021).

#### 2.2.1 Extraversion and neuroticism in driving contexts

- Extraversion: Characterized by sociability, talkativeness, and a proclivity for excitement-seeking. Empirical findings suggest that higher Extraversion often correlates with riskier driving styles, such as speeding, due to a stronger desire for stimulation (Thiffault and Bergeron, 2003; Taubman-Ben-Ari et al., 2004). Extraverted drivers also exhibit more casual attitudes toward road safety, which may reduce caution and increase mind-wandering (Tinella et al., 2022).
- Neuroticism: Reflects emotional instability, anxiety, and moodiness (Damon et al., 2006; Eysenck and Eysenck, 1975). In driving contexts, Neurotic individuals are prone to worry, frustration, and higher stress levels, often leading to poor concentration and more frequent errors or lapses (Biernacki and Lewkowicz, 2020). Their tendency toward mind-wandering can further heighten accident risk (Tinella et al., 2022).
- Characterized by hostility and coldness, this trait is often examined in large-scale personality surveys (Eysenck, 1983). Individuals scoring very low on Psychoticism may be described as having a “superego,” reflecting heightened regulation and conscience. Empirical findings suggest that higher levels of Psychoticism can predict exaggerated or incorrect reactions, particularly among older individuals (Biernacki and Lewkowicz, 2020).

**Figure 2:**
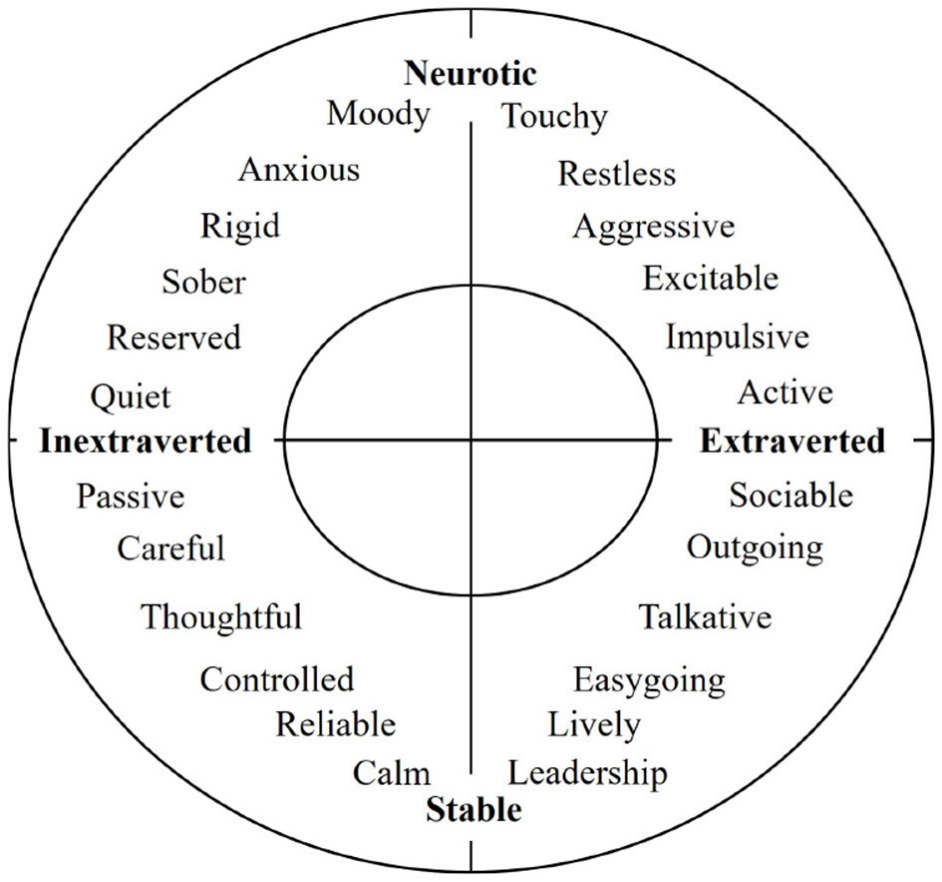
Two-dimensional model of extraversion and neuroticism (Eysenck, 1963).

In addition to the Eysenck P-E-N model, several other models have been put forward to delineate differences in human behavior based on individual traits. The Big Five personality model, for instance, encompasses multiple dimensions of personality, which include Extraversion, Neuroticism, Conscientiousness, Agreeableness, and Openness. While the Big Five model includes Conscientiousness, Agreeableness, and Openness, past research consistently points to Extraversion and Neuroticism as the traits most relevant to emotional reactivity and risky driving (Lajunen, 2001; Braitman and Braitman, 2017; Tinella et al., 2021). Specifically, Extraversion aligns with thrill-seeking and impulsivity, and Neuroticism aligns with anxiety and emotional volatility-both of which crucially affect on-road behaviors. In contrast, traits like Conscientiousness and Agreeableness, though important in broader psychological contexts, have demonstrated weaker or less direct links to immediate driving performance under arousal conditions. Figure 2 illustrates the structured higher-order traits within the Extraversion-Neuroticism two-dimensional model.

Extraversion and Neuroticism have a significant impact on emotional reaction, arousal states, and driving behaviors. These influences suggested individual differences and different driving styles under high arousal states. According to Tinella et al. (2022), individuals with high levels of Extraversion or Neuroticism are more prone to mind-wandering while driving, which has been linked to increased risky driving behaviors. This finding aligns with the Eysenck personality theory, which suggests that individuals with high levels of Extraversion exhibit a carefree and less inhibited disposition, potentially leading to more aggressive driving behaviors and a tendency to disregard regulations (Eysenck et al., 1985; Renner and Anderle, 2000). In the case of Neuroticism, individuals with high levels of Neuroticism tend to focus more on internal concerns and engage in attempts to address these concerns, resulting in frequent episodes of mind-wandering, as discussed in Robison et al. (2020). This points to the unstable internal state of Neurotic individuals, as also observed by Eysenck (1983). Wang and colleagues suggested in Wang et al. (2009) that Neuroticism and Extraversion have a strong connection with affect and emotion regulation. Their research revealed that Neuroticism exhibited a positive association with negative affect, with reappraisal (one of the emotion regulation strategies, alongside suppression) having a negative impact on this connection. Similar findings were observed in the relationship between Extraversion and positive affect, where Extraversion scores were positively associated with positive affect, and reappraisal mediated this connection in a positive manner.

Extraversion reflects individuals' inclination toward seeking adventure, and this inclination is significantly influenced by their subjective emotional states (Kuppens et al., 2017). Matthews (1987) suggested that the connections between Extraversion and arousal might be a result of their interaction. This interaction, especially when combined with impulsivity, can have a notable impact on one's performance. However, there are contrasting findings in some other studies, such as Andersson et al. (1970); Pestonjee and Singh (1980), which suggest an opposite direction, while some studies, like Perrine (1970); Wilson and Greensmith (1983), have found no significant results. This variability in findings may be attributed to the diversity of personality theories and questionnaires utilized. Given the intricate and occasionally conflicting results, it is plausible that Extraversion could exert an indirect influence and serve as a mediating factor in driving behaviors and decisions driven by emotions.

Demographic variables, such as age and gender, also influence Extraversion and Neuroticism. For example, Extraversion often increases from adolescence into early adulthood, while Neuroticism may decline over the lifespan (Viken et al., 1994). Such patterns imply that different age cohorts exhibit distinct personality profiles, which can lead to varied emotional and behavioral outcomes. Further, much of the existing research focuses on Western populations, suggesting a need for more culturally specific inquiries that might unveil novel dynamics in personality-emotion interactions, particularly in non-Western settings (Li et al., 2022).

Empirical findings also point to demographic nuances that can intersect with cultural context. For instance, older drivers high in Extraversion appear more prone to behaviors like talking on the phone while driving, suggesting that sociability or impulsivity may override caution (Parr et al., 2016). However, cultural norms around communication or technology use could modulate this relationship; in societies where phone use while driving is more socially accepted or less strictly enforced, highly Extraverted individuals might feel freer to engage in such risky activities. Cross-national data further indicate that countries with a higher proportion of highly Extraverted individuals experience elevated traffic fatalities (Lajunen, 2001), potentially reflecting both personality distributions and cultural attitudes toward risk-taking. Such findings underscore the importance of examining personality-emotion dynamics across diverse cultural settings, where different social norms and enforcement practices may either amplify or mitigate the impact of Extraversion on driving behaviors.

In sum, existing literature underscores Extraversion and Neuroticism as pivotal traits influencing emotional reactivity and driving behaviors. However, the interactive effects between these traits, high-arousal states, and external factors like age remain underexplored, particularly in culturally diverse contexts.

### 2.3 Driving behavior

Driving behavior encompasses the broad range of actions drivers take, including speed choices, acceleration patterns, steering control, and decisions about lane-changing or following distances (Toledo et al., 2007, 2009). These outcomes can be linked to deeper psychological factors, such as individual risk tolerance, emotional states, and personality.

Research consistently shows that speed-related metrics (e.g., acceleration rates, average speed, speed variability) are predictive of traffic accidents (af Wåhlberg, 2004, 2006a,b). A driver's willingness to accelerate beyond set limits or fluctuate speeds significantly may indicate higher sensation-seeking or emotional reactivity (Wasielewski, 1984). For instance, individuals high in Extraversion or Neuroticism may be more susceptible to driving faster when emotionally aroused, either out of impulsivity (Extraversion) or anxiety-driven impatience (Neuroticism). Moreover, studies in bus driving contexts have shown that acceleration is positively correlated with accident likelihood (af Wåhlberg, 2000). Translating these findings to private vehicle contexts suggests that personality traits and emotional states could jointly influence how aggressively or smoothly a driver accelerates under stress or excitement (Li et al., 2022).

The driver's steering wheel performance is another essential aspect of driving behaviors, as it directly influences the vehicle's lateral movement (Zhang et al., 2022a; Pawar and Velaga, 2021). The measurement of steering control includes steering speed and steering swerve. Factors such as age, gender, and driving experience have been found to impact steering wheel performance significantly. For example, older non-professional drivers showed better steering performance (lower standard deviation of speed) in low traffic flow conditions but worse performance in high traffic conditions (Chen et al., 2019). Female drivers were found to have a higher steering reversal rate (SRR) than male drivers in car-following situations (Pawar and Velaga, 2021). Additionally, the same study reported that each year of driving experience led to a significant improvement in steering stability control (SSC).

In addition to examining drivers' attitudes toward acceleration and speed in emotional states, it is essential to consider other driving behaviors that may indirectly be influenced by the driver's emotional state (Du et al., 2020; Zhang et al., 2022b). Factors such as head vehicle distance, fluctuations in lane maintenance, and eye fixation or gaze behavior play pivotal roles in shaping the overall driving experience and safety. Steinhauser et al. (2018) demonstrated that anger influenced both the average and the variability of the distance to the leading car. Nevertheless, in the angry condition, a positive impact on braking behavior was observed. Cai and Lin (2011) suggested that the arousal and valence levels have significantly influenced the eye's attention to the surrounding environment. Moreover, when drivers were in high arousal states, the lane deviation performance was more dynamic (Cai and Lin, 2011).

Collectively, past studies underscore the critical role of emotional valence and arousal in influencing how individuals drive. High-arousal negative emotions, like anger or anxiety, often correlate with aggressive or distracted behaviors (e.g., speeding, abrupt lane changes), while positive or low-arousal states can mitigate these tendencies. Personality traits-particularly Extraversion and Neuroticism-emerge as key moderators in this process, making some drivers more vulnerable to risk-taking under emotional strain. However, the interaction between (1) personality traits, (2) emotional states, and (3) demographic or cultural variables remains insufficiently explored. Most existing research is Western-centric, leaving gaps in understanding how these factors might play out in populations like Chinese drivers.

Addressing these research gaps is crucial for developing comprehensive models of emotional driving. Therefore, in the present study, we incorporate moderated moderation and mediated moderation analyses to examine how age, driving experience, and personality traits (Extraversion and Neuroticism) converge under different arousal states to shape critical driving behaviors (acceleration, speed stability, and steering). By tackling these understudied aspects in a Chinese driving context, we aim to deepen the field's understanding of cross-cultural factors and offer more robust insights into emotional driving phenomena.

### 2.4 Hypotheses

Emotions are widely acknowledged to influence driving behavior directly. However, their impact on the interplay between other human factors, such as personality traits and driver profiles, remains underexplored (Gross, 2013; Purnamaningsih, 2017). Building on prior research, we propose that Extraversion and Neuroticism may act as underlying factors shaping driving behaviors, particularly in high-arousal states (Song et al., 2021).

Drawing from studies on personality and emotion regulation, prior findings suggest that high-arousal states contribute to risky driving behaviors (Töre et al., 2023). However, it remains unclear how Extraversion and Neuroticism influence driving behaviors under different arousal conditions. Furthermore, driver profiles during emotional driving states have been largely overlooked. From a social psychology perspective, the influence of personality variations on driving performance in emotional states warrants attention.

Our study aims to uncover the psychological components underlying driving behaviors in high- and low-arousal situations. Specifically, we examine the relationships among drivers' profiles (age, driving experience), personality traits (Extraversion, Neuroticism), and driving behaviors (acceleration, speed stability, and steering performance).

Since driving experience does not show a clear causal relationship with personality traits, we focus on examining its moderation effects instead of mediation. This study primarily involves young and middle-aged Chinese drivers.^1^ Although gender is an important factor in traffic research, the sample's gender imbalance limits analysis of gender differences; therefore, it will be included as a covariate to reflect real-world traffic dynamics.

1. **Age and personality traits**
  Developmental psychology studies suggest that older drivers exhibit higher Extraversion and lower Neuroticism due to personality maturation and changes in social environments (Hutteman et al., 2014; Caspi and Shiner, 2007; Turner, 1988). In the Chinese context, these patterns are further supported by findings on age-related personality shifts ([LI Qi-Meng, 2015).
  - H1.1: Age is positively associated with Extraversion.
  - H1.2: Age is negatively associated with Neuroticism.
2. **Driver profiles and driving behaviors**
  Previous studies have shown that older and more experienced drivers tend to adopt safer driving behaviors, such as reduced acceleration and greater speed stability (Witt et al., 2019; Mouloua et al., 2007; Tao et al., 2017). Conversely, personality traits such as Extraversion and Neuroticism are linked to riskier and more unstable behaviors (Renner and Anderle, 2000).
  - H2.1: Higher age is associated with reduced acceleration, improved speed stability, and enhanced steering capabilities.
  - H2.2: Greater driving experience is associated with reduced acceleration, improved speed stability, and enhanced steering capabilities.
  - H2.3: Extraversion is associated with higher acceleration and poorer speed stability and steering stability.
  - H2.4: Neuroticism is associated with higher acceleration and poorer speed stability and steering stability.
3. **Personality as a mediator/moderator**
  The role of personality traits in mediating or moderating the relationships between age, driving experience, and driving behaviors reflects the nuanced ways in which personality influences behavior across contexts (Renner and Anderle, 2000).
  - H3.1: Extraversion mediates the relationship between age and driving behaviors.
  - H3.2: Neuroticism mediates the relationship between age and driving behaviors.
  - H3.3: Extraversion moderates the relationship between driving experience and driving behaviors.
  - H3.4: Neuroticism moderates the relationship between driving experience and driving behaviors.
4. **Arousal state as a moderator**
  High-arousal states have been shown to exacerbate risky behaviors (Gross, 2013; Song et al., 2021), potentially moderating the influence of personality traits on driving behaviors.
  - H4: Arousal states moderate the relationship between personality traits and driving behaviors.

## 3 Methods

### 3.1 Participants

**Figure 3:**
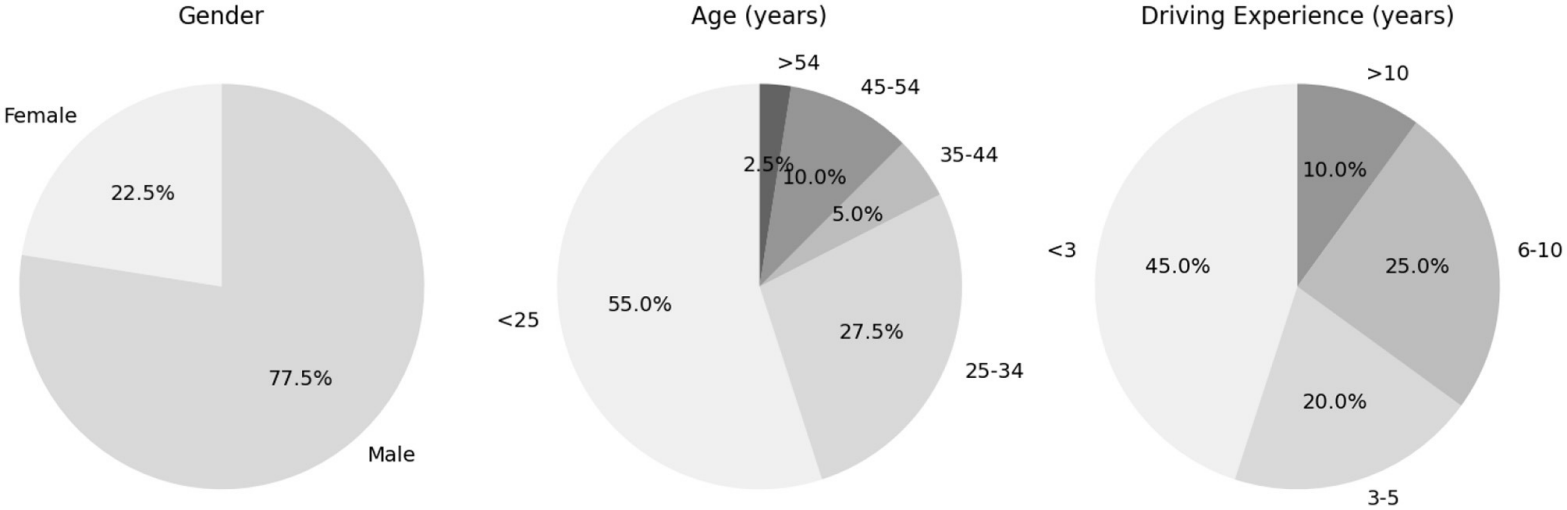
Socio-demographic characteristics of participants.

The data was collected from a multi-modal human emotions driving study described in Li et al. (2022). The dataset comprised 40 drivers from China, of which 31 were male and 9 were female. All participants' data was used in this paper. The mean age of the 40 participants was 28.1 years with a standard deviation of 9.47 years. Figure 3 provides details on the demographic characteristics. On average, the drivers had 8.93 years of driving experience. According to the Traffic Management Bureau of the Public Security Ministry, by the end of 2022, China had 481 million car drivers, of whom 162 million were female-indicating a male-to-female ratio of roughly 2:1 (Song et al., 2024). Although our sample shows a somewhat higher ratio of male to female participants (about 3.4:1), it still partially reflects the overall gender distribution of drivers in China.

To ensure safety and reliability in the driving tasks, all participants were screened for normal or corrected vision and hearing and reported their health status, including any medical conditions, prior to the experiment. Additionally, participants were instructed to maintain a regular 24-h schedule, abstain from alcohol and stimulating substances before the experiment, and meet the inclusion criteria of having at least one year of driving experience with a valid driver's license. Each person was rewarded 200 RMB after experiments.

### 3.2 Apparatus and stimuli

The simulator is a fixed-based driving simulator and is designed to provide a 270° field of view for the driver, with a half-cab platform and an automatic transmission. It includes a simulated rear-view mirror that allows the driver to keep an eye on the traffic behind them. In addition, the simulator has speakers that produce the sounds of the engine and ambient noise, as well as a woofer that replicates the vibration of the vehicle. The dashboard of the simulator is displayed on an LCD screen with a high resolution of 1,920 × 720 and a refresh rate of 60Hz. This screen displays car state parameters such as the speed and gear position.

**Figure 4:**
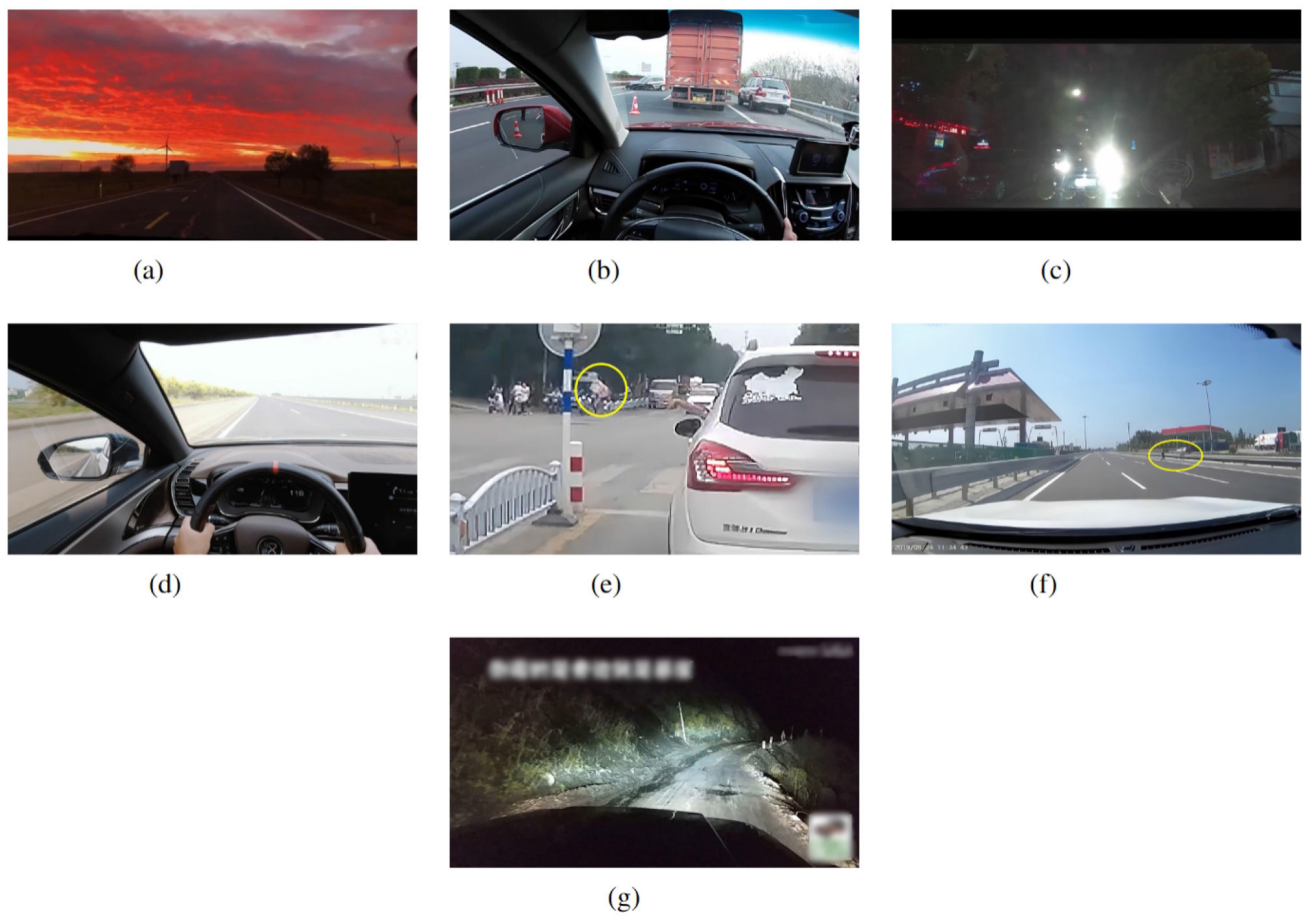
Examples of emotional stimulus video clips. The scenes in the study are designed to elicit specific emotional responses. **(A)** The “Happiness” scene portrays a driver enjoying a pleasant drive on a quiet road, accompanied by music and the approaching dusk (clip lasts 28 seconds). In contrast, **(B)** the “Sadness” scene depicts a driver witnessing a terrible car accident, evoking feelings of sadness (clip lasts 15 seconds). **(C)** The “Anger” scene displayed a driver navigating through a town when a car traveling in the same lane but in the opposite direction. This car forces the driver to stop rudely instead of yielding. The approaching car keeps its high beams on, severely impairing the driver's visibility (clip lasts 11 seconds). **(D)** The “Neutral” scene portrays a driver on an empty highway on a sunny day (clip lasts 29 seconds). **(E)** The “Disgust” scene involves a driver noticing the car in front littering by throwing a plastic bottle out of the window. The impolite behavior makes the driver feel disgusted (clip lasts 11 seconds). **(F)** The “Surprise” scene features a driver observing a pedestrian crossing over a highway barrier. The speed of the car is high, and the pedestrian's behavior makes the driver very surprising (clip lasts 10 seconds). **(G)** The “Fear” scene describes a driver navigating a cliff-edge road at night with its wheels stuck in the mud while the passenger screaming in terror (clip lasts 25 seconds).

The driving scenario consisted of a two-lane, two-way highway route with a total length of 3 km. Participants were instructed to drive in the right lane without changing lanes while maintaining a constant speed of 80 km/h. The oncoming road had a density of 3 cars per kilometer, and there were an average of 2 buildings per kilometer along the traffic route. The experiment employed a 20-inch central display simulator with a resolution of 1,280 × 1,024 and a refresh rate of 60Hz to present audio-video stimuli. The stimuli consisted of 7 videos, namely neutral, surprise, disgust, sadness, happiness, fear, and anger. Previous studies have confirmed the effectiveness of these videos in eliciting emotional arousal among drivers (Li et al., 2022). For example, the fear stimulus video depicted a car driving along the edge of a cliff in darkness, with its wheels stuck in the mud while the passengers made frightening noises after the car slowly began to move forward. All emotional stimulus video clips are shown in Figure 4. For each emotion stimulus, there is only one video clip. The procedure required drivers to first watch a video in its entirety and then commence their driving task. During the driving, the same video segment continued to play on a loop to maintain a consistent level of stimulation.

These emotions can highly reflect humans' emotional spectrum. In Eysenck's theory (Eysenck, 1983), neutral emotion is characterized by a lack of strong feelings or emotional intensity. It typically falls in the middle of the valence and arousal spectrum. Surprise is characterized by its high level of arousal. It can be either a positive or negative emotion, depending on the context. It often involves a sudden and intense reaction to unexpected events. Disgust is moderately arousing and is consistently negative in valence. It involves a sense of revulsion or repulsion, often triggered by something offensive or unpleasant. Sadness can vary in arousal from mild to moderate and is characterized by negative valence. It involves feelings of unhappiness, sorrow, and a lack of energy. Happiness can range from low to high arousal levels, and it is consistently positive in valence. It encompasses feelings of joy, contentment, and well-being. Fear is highly arousing and has a negative valence. It involves a strong emotional reaction to perceived threats or danger. Anger is highly arousing and carries a negative valence. It involves strong feelings of frustration and irritation. These abundant emotions help us to select high arousal state data.

### 3.3 Measures

**Figure 5:**
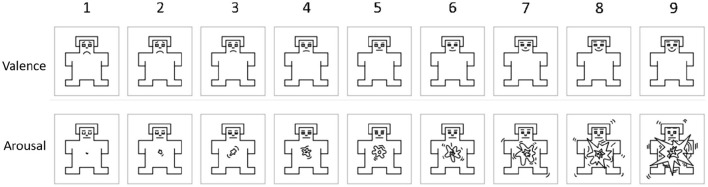
The self-assessment manikin (SAM) (Bradley and Lang, 1994).

The participants' emotional state was evaluated based on two dimensions: valence and arousal. The Self-Assessment Manikin (SAM) no-verb questionnaire (Bradley and Lang, 1994) was employed for this purpose. SAM is frequently used in conjunction with measurements of driving behaviors or physiological indicators to obtain a comprehensive understanding of emotional responses. The Self-Assessment Manikin (SAM) scale comprises three sets of figures, each representing a continuum along one of three affective dimensions: Valence, Arousal, and Dominance. In our analysis, we primarily focused on the Valence and Arousal dimensions, as these are more commonly used axes, while the Dominance dimension was not included. The Valence scale is depicted by a range of figures expressing emotions from smiling and happy to frowning and unhappy. Similarly, the Arousal scale is illustrated with figures ranging from excited and wide-eyed to relaxed and sleepy. As illustrated in Figure 5, the valence range spans from 1 (extremely unpleasant) to 9 (extremely pleasant), while the arousal range ranges from 1 (extremely excited) to 9 (extremely unexcited). Participants indicate their emotional state in response to a stimulus by selecting the figure that best represents their experience on each dimension. SAM's visual and intuitive format makes it particularly useful for studies involving participants with varying levels of literacy or language proficiency (Khosrowabadi et al., 2010; Koch et al., 2017).

Both qualitative and quantitative measures were recorded. After experiencing different driving conditions in a random order of a total of 7 videos, the participants selected their level of valence and arousal based on their subjective feelings. SAM was utilized to measure the participants' level of arousal and valence, with a scale ranging from 1 to 9. Moreover, the Eysenck Personality Questionnaire (EPQ) is used to measure drivers' personality traits (Eysenck and Eysenck, 1975). The EPQ used in this experiment includes 88 questions (Li et al., 2022) to access 4 dimensions of personality: E-Extraversion, N-Neuroticism, P-Psychoticism, and L-Lie. The questionnaire consists of a series of yes-no questions, each aimed at measuring specific aspects of these personality traits. Extraversion evaluates sociability and impulsiveness. Neuroticism is characterized by elevated levels of anxiety and depression, while Psychoticism is often associated with tough-mindedness and anger. Additionally, insincerity (i.e., L-Lie) is used to evaluate the validity and accuracy of the results. Our research primarily investigates the trait of Extraversion, often linked with positive emotions. Additionally, we examine Neuroticism, a trait typically associated with emotional stability and the propensity for anxiety. Psychoticism is not included in our analysis. The EPQ is renowned for its psychometric properties, including reliability and validity, making it a popular choice for personality research in various fields (Taubman-Ben-Ari et al., 2004; Du et al., 2018).

In addition, the simulator recorded multiple time series of car parameters, including acceleration, lateral acceleration, gas pedal position degree, brake pedal force, steering wheel position, and speed. Consistent with prior research, we focused on acceleration (af Wåhlberg, 2000), speed (Toledo et al., 2009), and steering wheel (Chen et al., 2019) as key indicators to represent the driving behavior and attitude. To represent acceleration behavior, we used the 95th percentile resulting acceleration, denoted as *ACC*_95*th*_. Furthermore, we calculated the standard deviation of speed (*Speed*_*std*_) as an indicator of speed stability, and the standard deviation of steering wheel speed (*Steering*_*std*_) as an indicator of steering performance. At last, we applied log to all the driving behavior data. More details are provided in Section 3.5.

### 3.4 Experimental process

In this experiment, all participants were involved, and they were given instructions to maintain a regular 24-h schedule and avoid consuming alcohol or stimulating drugs before the experiment. Upon arriving at the testing place, each participant completed a demographic form and a health form to check their health state and to ensure they were fit for the experiment. The health form included requirements about health history, medication use, mental status, etc. After being equipped with the EEG device,^2^ the participants were given a 10-minute training session to get used to the simulator environment and the signal recording process. The training took place on an 8 km stretch of highway with four lanes in both directions, where the drivers were asked to comply with the traffic laws. After the training, the participants completed a Simulator Sickness Questionnaire (SSQ) to ensure that the participants had no mental comfort like being fatigued or a physical illness (Singla et al., 2021). Following the training and information collection session, participants completed 7 emotional driving experiments in random order: neutral, surprise, disgust, sadness, happiness, fear, and anger. The driving scenario was a 3 km straight section of road with two lanes and oncoming traffic at a rate of 3 vehicles per kilometer. The road included 2 buildings in the field of vision every kilometer, and the weather was sunny with good visibility. Participants were instructed to drive at a speed of approximately 80 km/h in the right lane with no vehicles in front or behind. The 7 emotional induction materials were selected based on their efficiency as reported in Li et al. (2022). Participants were instructed to watch video and audio clips displayed on the screen in a random manner while maintaining emotional control as if they were driving.

After watching the videos, participants began driving, and data recording started. Following each emotional induction and driving session, participants completed the Differential Emotion Scale (DES) and the Self-Assessment Manikin (SAM). The DES, a multi-dimensional self-report questionnaire designed to assess individuals' emotional experiences (Izard and Izard, 1977), was employed to confirm the elicitation and intensity of specific discrete emotions (e.g., anger, sadness, surprise). The recorded data were considered valid only when the emotions reported on the DES aligned with those intended by the video segments. The SAM, on the other hand, was used to measure participants' Valence and Arousal levels-two core dimensions of emotional states that are widely studied in traffic psychology due to their direct influence on cognitive processes and driving behaviors (Du et al., 2020; Ünal et al., 2013). The Dominance dimension of SAM was excluded from this study, as it is less commonly included in driving-related research and was not directly relevant to the study's objectives.

**Figure 6:**
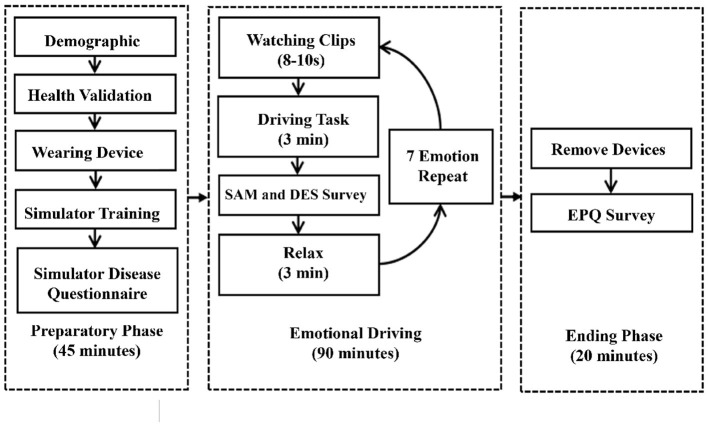
Experiment process.

Following each driving task, participants were given a 3-min pause to relax and regain a neutral state before encountering the next emotional stimulus. At the end of the experiment, participants removed the devices, left the simulator, and completed the EPQ survey. The experimental process is depicted in Figure 6. Two hundred and forty emotional driving experiments were validated (i.e., this included all 40 participants recordings). Forty emotional driving experiments were deleted because based on the results of DES, certain emotions were not shown as expected. The discrete emotion data from DES were not directly linked to the Valence and Arousal dimensions of SAM and were therefore not used in this study's analysis.

### 3.5 Data processing

**Figure 7:**
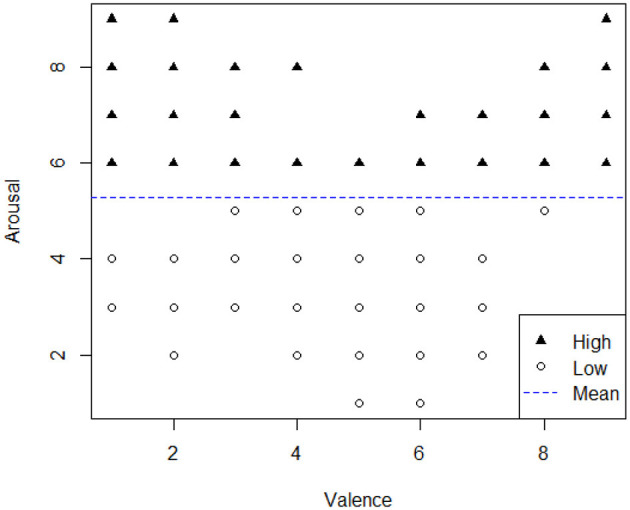
Visualization of arousal states. The arousal and valence of all experimental samples are mapped in this figure. Because there are only 9 scales on the horizontal and vertical coordinates, most of the points overlap in the figure. The whole sample is divided into high and low arousal, according to the mean value of data, with black triangles (5.3 < *arousal* ≤ 9) and Low arousal with white triangles (1 ≤ *arousal* < 5.3). The blue line segment represents the average value of arousal.

First, the participants' levels of arousal and valence were measured using the Self-Assessment Manikin (SAM) scale, which ranges from 1 to 9. The arousal states were categorized as high and low. We categorized instances where the arousal level exceeded the mean arousal of the samples (i.e., mean arousal = 5.3) as high arousal states, while the others were considered low arousal states. This approach not only aligns with prior studies such as Du et al. (2020), but it also simplifies the interpretation of emotional states, making the distinction between high-intensity (e.g., happy, angry) and low-intensity (e.g., sad, boring) emotions clearer and more actionable for analysis (see Figure 7).

The high arousal samples consist of 132 instances and the number of low arousal samples is 108 (i.e., from all male and female drivers), each containing questionnaire data, and multiple time series driving data collected during one experiment. As previously mentioned, the demographic characteristics used for analysis include age, and driving experience, which were obtained from the demographic questionnaire. The personality characteristics used in the analysis are Extraversion (E) and Neuroticism (N), as accessed by the EPQ. Driving behavior data was captured using the simulation equipment, with the original recordings containing parameters such as acceleration, speed, and steering wheel position. To stabilize the variance and handle heteroscedasticity, we employed log transformation on the output variables of acceleration, velocity stability, and steering stability. The log*ACC*_95*th*_ (acceleration choice) was directly calculated using Equation 1. For each experiment, log*Speed*_*SD*_ (speed stability) was calculated using Equation 2 from the driving data. log*Steering*_*SD*_ (steering performance) was obtained from the steering wheel position using Equations 3, 4. In the formulas, *i* represents the *i*_*th*_ moment in driving, and the *Timeinterval* represents the sampling interval of the simulator: 0.33 seconds.


(1) $$\begin{array}{l} \log ACC_{95th}=\log_{10}\left(\underset{95\%}{\max}ACC+1\right) \end{array}$$

(2) $$\begin{array}{l} \log Speed_{SD}=\log_{10}(\sqrt{\frac{\sum {\left(Speed_{i}-Sp\bar{e}ed_{i}\right)}^{2}}{n-1}}+1) \end{array}$$

(3) $$\begin{array}{l} Steeringspeed_{i}=\frac{{\text{Steering position}}_{i+1}-{\text{Steering position}}_{i}}{\text{Timeinterval}} \end{array}$$

(4) $$\begin{array}{l} \log Steering_{SD}=\log_{10}(\sqrt{\frac{\sum {\left(Steering\;speed_{i}-Steerin\bar{g}\;speed_{i}\right)}^{2}}{n-1}}+1) \end{array}$$

### 3.6 Moderated mediation and moderated moderation models

To investigate the complex relationships between personality traits, driving experience, arousal states, and driving behaviors, we employed moderated mediation and moderated moderation models using Hayes's PROCESS macro.

#### 3.6.1 Moderated mediation

Moderated mediation examines whether the indirect effect of an independent variable (e.g., Age) on a dependent variable (e.g., acceleration) through a mediator (e.g., Extraversion) varies across levels of a moderator (e.g., Arousal States). This approach allows us to explore how situational or individual factors influence the pathways linking predictors and outcomes. For this study, we tested moderated mediation effects using Hayes's PROCESS Model 14. Hypotheses H3.1 and H3.2 were framed to investigate whether Extraversion and Neuroticism mediated the relationship between Age and driving behaviors, and whether these indirect effects were moderated by Arousal States (H4).

#### 3.6.2 Moderated moderation

Moderated moderation evaluates whether the interaction effect between two variables (e.g., Driving Experience and Extraversion) on an outcome (e.g., Speed Variability) is further moderated by a third variable (e.g., Arousal States). This model helps capture multi-level interactions that shape behavioral outcomes. In this study, we tested moderated moderation effects using Hayes's PROCESS Model 3. Hypotheses H3.3 and H3.4 focused on whether Extraversion and Neuroticism moderated the relationship between Driving Experience and driving behaviors, with additional exploration of Arousal States as a second moderator.

#### 3.6.3 Model rationale and application

The application of these models was guided by the study's hypotheses, aiming to provide a comprehensive understanding of how personality traits and situational factors interact to influence driving behaviors. Moderated mediation models were used to evaluate conditional indirect effects, capturing both personality-mediated pathways and their moderation by emotional states. Moderated moderation models were employed to assess three-way interactions among predictors, moderators, and outcomes.

#### 3.6.4 Software and analytical approach

All analyses were conducted using Hayes's PROCESS macro within the R environment. PROCESS Model 14 was used for moderated mediation analyses, while PROCESS Model 3 was employed for moderated moderation analyses. Bootstrapping with 10,000 samples was applied to estimate confidence intervals, ensuring robustness of the findings. Significance levels, confidence intervals, and effect sizes were reported to provide a comprehensive interpretation of the results.

These models allowed us to test both direct and conditional indirect effects, aligning the analysis framework with the hypotheses outlined in this study. Detailed results are presented in the respective sections of this manuscript.

## 4 Analysis

**Figure 8:**
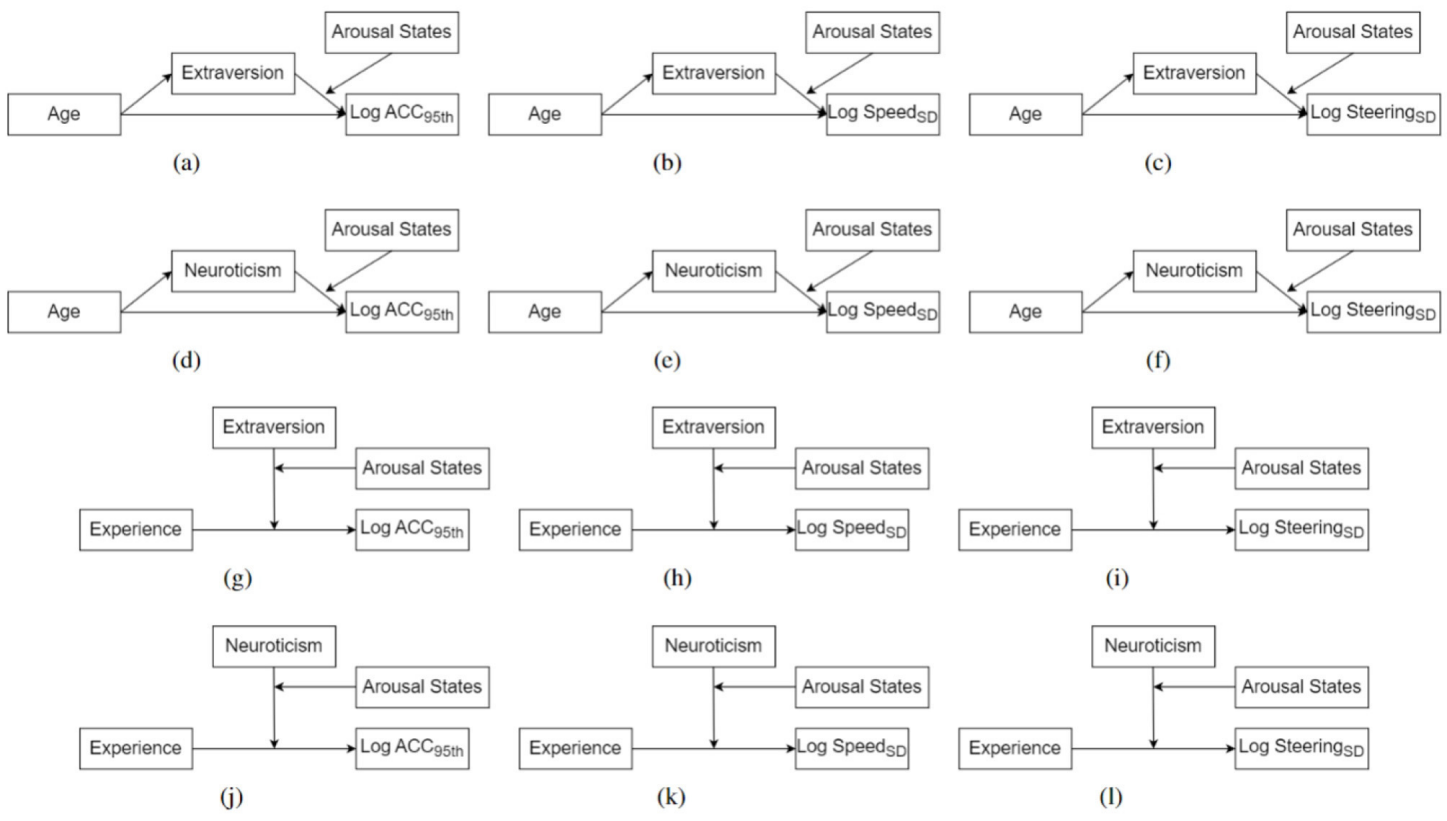
The hypothesized moderated moderation and moderated mediation models. **(A)** Model 1. **(B)** Model 2. **(C)** Model 3. **(D)** Model 4. **(E)** Model 5. **(F)** Model 6. **(G)** Model 7. **(H)** Model 8. **(I)** Model 9. **(J)** Model 10. **(K)** Model 11. **(L)** Model 12.

We used the PROCESS package with R to perform multiple moderation and mediation analyses to investigate our hypothesis and the causality between different elements (Hayes, 2017). The moderated mediation effect was analyzed by PROCESS Model 14. The moderated moderation effect was analyzed by PROCESS Model 3. The gender was treated as covariant in our models. The basic predictors included age, personality traits, and driving experience. Based on our literature review and hypothesis (see Sections 1, 2), we considered Extraversion and Neuroticism personality traits as mediators in the relationship between driver age and driving behaviors. They also performed the moderator role in the relation between driving experience and driving behaviors, as mentioned in Section 2.4. The moderated moderation and moderated mediation effect of arousal states on the relation between personality and driving behaviors are considered. The driving behaviors were measured by log*ACC*_95*th*_, log*Speed*_*std*_, and log*Steering*_*SD*_. The model diagrams corresponding to our hypothesis are shown in Figure 8.

Bootstrapping was performed with 10,000 resamples (boot = 10,000) to provide robust estimates of confidence intervals, as recommended for moderated mediation analyses. The model parameters were set to include both first-order and second-order moments (moments = 2) for detailed variance calculations. A fixed random seed (seed = 654,321) was specified to ensure replicability of the results (Hayes, 2017).

The model 1–3 was designed according to H1.1, H2.1, H2.3, H3.1, and H4. The model 4–6 was designed according to H1.2, H2.1, H2.4, H3.2, and H4. The model 7-9 was designed according to H2.2, H3.3, and H4. The model 10–12 was designed according to H2.2, H3.4, and H4. Before the analysis, we used the Shapiro-Wilk test to test for the normality of data. The Shapiro-Wilk test showed that age (*w* = 0.9, *p* < 0.05), driving experience (*w* = 0.7, *p* < 0.05), Extraversion (*w* = 0.9, *p* < 0.05), Neuroticism (*w* = 0.9, *p* < 0.05), log*ACC*_95*th*_ (*w* = 0.9, *p* < 0.05), log*Speed*_*std*_ (*w* = 0.8, *p* < 0.05), and log*Steering*_*SD*_ (*w* = 0.8, *p* < 0.05) did not satisfy the normal distribution. However, the PROCESS tool does not assume a normal distribution of data, so our analysis remains reliable (Hayes, 2017). Additionally, we employed bootstrapping methods in our analysis.

To assess multicollinearity, VIF tests were performed on each models. The VIF values for each model were well below the threshold of 1.5, indicating no significant multicollinearity among the predictors. These results demonstrate that multicollinearity is not a concern in either model.

## 5 Experimental results

### 5.1 Moderated mediation analysis with extraversion

The moderated mediation models with Extraversion as a mediator were tested using Hayes's PROCESS macro (model 14), as shown in Figures 7A–C. These models explored the relationships between Age, Extraversion, arousal states, and driving behaviors, alongside the covariate effects of Gender.

The results supported **H1.1**, demonstrating that Age positively predicted Extraversion (β = 0.113, *p* < 0.001), indicating that older drivers tend to exhibit higher levels of Extraversion. Furthermore, Gender significantly influenced Extraversion (β = −3.872, *p* < 0.001), with male drivers (coded as 1) scoring higher than female drivers (coded as 2). Together, Age and Gender accounted for 20.6% of the variance in Extraversion (*R*^2^ = 0.206, *F* = 30.722, *p* < 0.001).

In support of **H2.1**, Age negatively predicted acceleration (log ACC_95_ : β = −0.014, *p* < 0.001) and speed variability (log Speed_std_ : β = −0.006, *p* < 0.001), while positively predicting steering variability (log Steering_std_ : β = 0.009, *p* < 0.001). These results suggest that older drivers tend to drive more cautiously, with reduced variability in acceleration and speed but slightly increased variability in steering. Gender also influences driving behaviors. Male drivers exhibited greater acceleration (log ACC_95_ : β = −0.075, *p* < 0.05), although no significant effects of Gender were observed for speed or steering variability (log Speed_std_ : β = −0.003, *p* = 0.932; log Steering_std_ : β = −0.019, *p* = 0.542).

The results further supported **H2.3** and **H3.1**, as Extraversion mediated the relationship between Age and driving behaviors. Specifically, Extraversion was positively associated with acceleration (log ACC_95_ : β = 0.013, *p* < 0.001) and speed variability (log Speed_std_ : β = 0.010, *p* < 0.001), aligning with theories that Extraverted individuals are more stimulation-seeking and risk-prone. However, Extraversion did not significantly affect steering variability (log Steering_std_ : β = 0.003, *p* = 0.278).

Moderated mediation analysis revealed that arousal states amplified the indirect effects of Extraversion on driving behaviors, supporting **H4**. In high-arousal states, the interaction between Extraversion and arousal significantly increased the indirect effect of Age on acceleration [$\Delta R^{2}=0.014,F_{\left(1,\;234\right)}=4.431,p<0.05$] and speed variability [$\Delta R^{2}=0.020,F_{\left(1,\;234\right)}=5.261,p<0.05$]. For log ACC_95_, the pre-bootstrap conditional indirect effect was 0.002 (*SE* = 0.0006, 95% CI = [0.0007, 0.0033]), with post-bootstrap results refining this to a 95% CI of [0.0011, 0.0031]. Similarly, for log Speed_std_, the pre-bootstrap conditional indirect effect was 0.0017 (*SE* = 0.0005, 95% CI = [0.0009, 0.0025]), while the bootstrapped results narrowed this to [0.0007, 0.0029].

**Figure 9:**
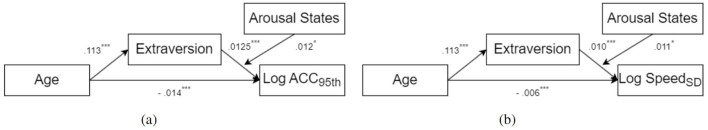
The moderated mediation model. Statistically significant relationships are retained by solid lines. The coefficients are shown next to each regression path. ^*^ indicates *p* < 0.05, ^***^ indicates *p* < 0.001. **(A)** Model 1. **(B)** Model 2.

**Figure 10:**
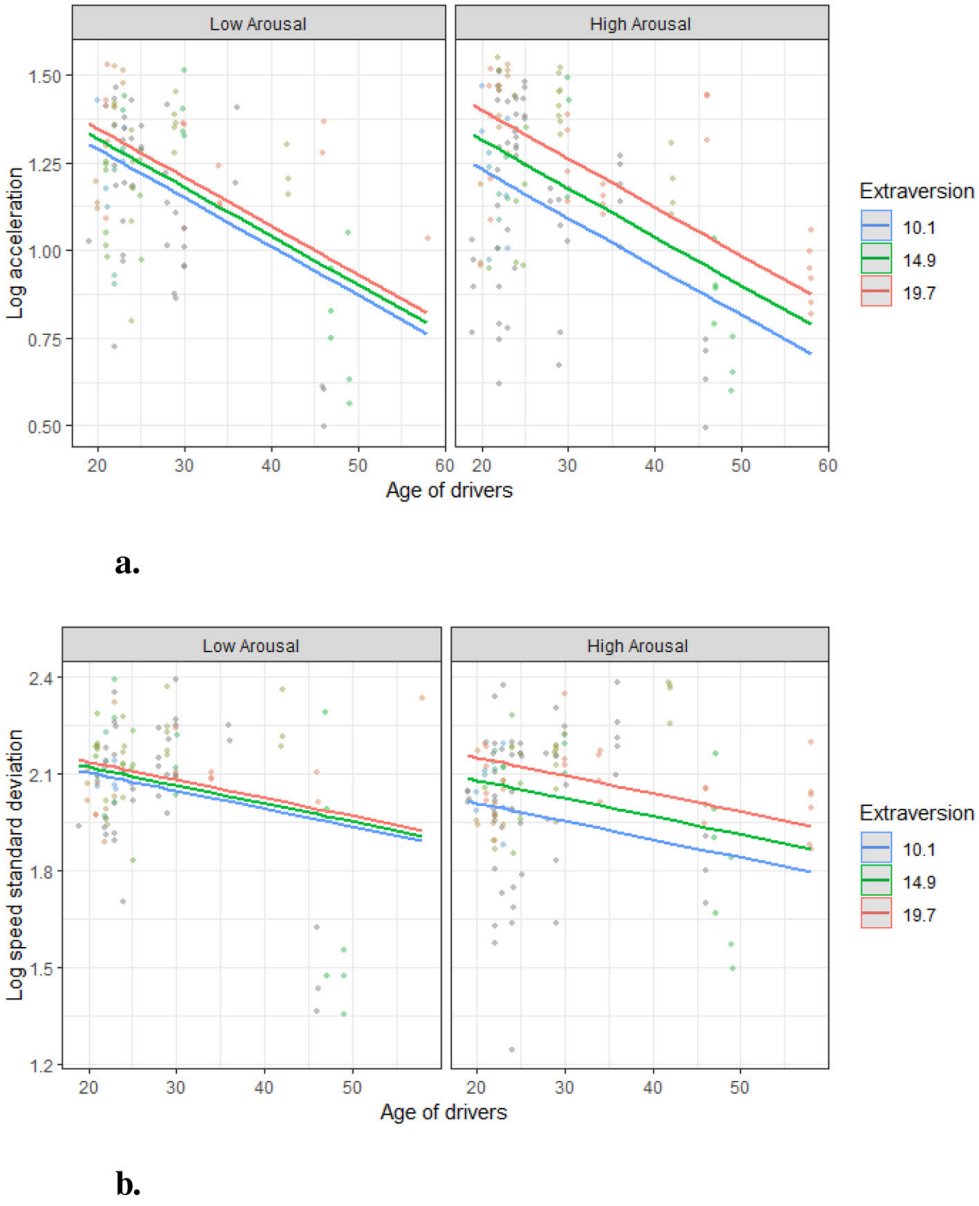
Mediation effect of Extraversion and moderated effect of arousal states. The Extraversion levels were grouped by the mean value and +/−1 standard deviation. **(A)** Moderated mediation effect on acceleration. **(B)** Moderated mediation effect on speed standard deviation.

These findings highlight the critical role of emotional states, particularly high arousal, in amplifying the influence of Extraversion on driving behaviors. High-arousal conditions were found to significantly increase variability in both acceleration and speed, emphasizing the dynamic interplay between personality traits and situational factors in shaping driving patterns. The detailed regression results (models 1–3), which showcase these interactions, are presented in Table 1. Summaries of the indirect effects for models 1 and 2 are provided in Table 2. Furthermore, a schematic diagram illustrating the moderated mediation relationships is shown in Figures 9, 10A offers a comprehensive visualization of the interaction among age, Extraversion, arousal states, and driving behaviors under various conditions.

**Table 1:** Moderated mediation analysis when Extraversion as mediator.

	**E (M)**		**Log ACC95 (Y1)**		**Log Speed SD (Y2)**		**Log Steering SD (Y3)**	
**Predictor**	**β**	***t***	**β**	***t***	**β**	***t***	**β**	***t***
Age	0.113^***^	0.995	–0.014^***^	-9.379	–0.006^***^	–4.225	0.009^***^	6.306
G	–3.872^***^	-5.361	–0.075^*^	-2.084	–0.003	–0.085	–0.019	–0.542
E (M)	-	-	0.013^***^	4.081	0.010^***^	3.538	0.003	1.088
A (W)	-	-	–0.0032	–0.121	–0.041	–1.729	0.004	0.177
M*W	-	-	0.012^*^	2.105	0.011^*^	2.294	–0.005	–0.902
*R* ^2^	0.206	-	0.288	-	0.120	-	0.200	-
*F*	30.722	-	18.897	-	6.382	-	11.677	-

E, Extraversion; A, Arousal states; M, Mediator; W, Moderator in mediation; Y1, output 1; Y2, output 2; Y3, output 3; Significance level:

* *p* < 0.05;

*** *p* < 0.001.

**Table 2:** Conditional indirect effect of age on driving behaviors through Extraversion.

**Arousal States**	**Indirect Effect**	**Pre-Boot CI**	**Post-Boot CI**
**Log ACC95 (Y1)**			
Low	0.0007	(–0.0002, 0.0015)	(–0.0003, 0.0012)
High	**0.002**	(0.0007, 0.0033)	(0.0011, 0.0031)
**Log Speed SD (Y2)**			
Low	0.0004	(–0.0002, 0.0015)	(–0.0003, 0.0012)
High	**0.0017**	(0.0009, 0.0025)	(0.0007, 0.0029)

CI, Confidence Interval; Pre-Boot, Confidence interval before bootstrapping; Post-Boot, Bootstrapped confidence interval. Bold values indicate a significant effect.

### 5.2 Moderated mediation analysis with neuroticism

The moderated mediation models with Neuroticism as a mediator were tested using Hayes's PROCESS macro (model 14), as shown in Figures 8D–F.

The models results showed that both **H1.2** and **H3.2** were not supported. While Age positively predicted Neuroticism (β = 0.046, *t* = 1.138, *p* = 0.256), this effect was not statistically significant. In contrast, Gender significantly predicted Neuroticism (β = 2.524, *t* = 2.642, *p* < 0.01), with female drivers (coded as 2) scoring higher than male drivers (coded as 1). Together, Age and Gender explained 2.9% of the variance in Neuroticism (*R*^2^ = 0.029, *F* = 3.540, *p* < 0.05). Consequently, since the path from Age to Neuroticism was not significant, Neuroticism could not act as a mediator, thereby invalidating its role in supporting **H3.2**.

In line with **H2.1** and results in Section 5.1, age negatively predicted acceleration (log ACC_95_ : β = −0.012, *t* = −8.020, *p* < 0.001) and speed variability (log Speed_std_ : β = −0.005, *t* = −3.433, *p* < 0.001), while positively predicting steering variability (log Steering_std_ : β = 0.009, *t* = 6.690, *p* < 0.001). Gender negatively predicted acceleration (log ACC_95_ : β = −0.111, *t* = −3.057, *p* < 0.01), indicating less stable acceleration patterns among male drivers.

**H2.4** was partially supported. Neuroticism was positively associated with speed variability (log Speed_std_ : β = 0.005, *t* = 2.296, *p* < 0.05), suggesting that individuals with higher levels of Neuroticism experience greater speed fluctuations. However, Neuroticism did not significantly affect acceleration (log ACC_95_ : β = −0.002, *t* = −0.894, *p* = 0.372) or steering variability (log Steering_std_ : β = 0.002, *t* = 1.024, *p* = 0.307).

**H4** was not supported when neuroticsim played a mediated role. Arousal states did not significantly moderate the relationship between Neuroticism and driving behaviors. The interaction term between Neuroticism and arousal states was not significant for acceleration (β = −0.006, *t* = −1.154, *p* = 0.250), speed variability (β = 0.002, *t* = 0.457, *p* = 0.648), or steering variability (β = −0.006, *t* = −1.386, *p* = 0.167).

These findings highlight that, unlike Extraversion, Neuroticism plays a more limited role in influencing driving behaviors. However, its positive association with speed variability suggests its relevance for understanding individual differences in driving performance. The detailed regression results are presented in Table 3.

**Table 3:** Moderated mediation analysis when neuroticism as mediator.

	**N (M)**		**Log ACC95 (Y1)**		**Log Speed SD (Y2)**		**Log Steering SD (Y3)**	
**Predictors**	**β**	***t***	**β**	***t***	**β**	***t***	**β**	***t***
Age	0.046	1.138	–0.012^***^	–8.020	–0.005^***^	–3.4331	0.009^***^	6.690
Gender	2.524^***^	2.642	–0.111^**^	–3.057	–0.054	-1.669	–0.019	–0.542
N (M)	-	-	–0.002	–0.894	0.005^*^	2.296	0.002	1.024
A (W)	-	-	–0.002	–0.063	–0.037	-1.528	0.006	0.249
M * W	-	-	–0.006	–1.154	0.002	0.457	–0.006	–1.386
*R* ^2^	0.029	-	0.233	-	0.078	-	0.203	-
*F*	3.540	-	14.201	-	3.935	-	11.948	-

N, Neuroticism; A, Arousal states; M, Mediator; W, Moderator in mediation; Y1, output 1; Y2, output 2; Y3, output 3; Significance level:

* *p* < 0.05;

** *p* < 0.01;

*** *p* < 0.001.

### 5.3 Moderated moderation analysis with extraversion

The moderated moderation models were established as shown in Figures 8G–L, which were tested using Hayes's PROCESS macro (model 3). These models explored the relationships between Driving Experience, Extraversion, Neuroticism, arousal states, and driving behaviors, alongside the covariate effects of Gender. However, no significant moderation effects of arousal states were observed in any of the models. Consequently, the focus shifted from the moderated moderation effects to identifying the moderation effects, which revealed a significant moderating role of Neuroticism in the relationship between Driving Experience and driving behaviors.

The results showed that **H3.3** was not supported. Specifically, the interaction term between Driving Experience and Extraversion (X*M) did not significantly predict acceleration (log ACC_95_ : β = 0.001, *t* = 0.359, *p* = 0.720), speed variability (log Speed_std_ : β = 0.002, *t* = 1.21, *p* = 0.227), or steering variability (log Steering_std_ : β = −0.000, *t* = −0.006, *p* = 0.995). Similarly, **H4**, which hypothesized a moderated moderation effect, was not supported, as the three-way interaction term (X*M*Z) was non-significant across all driving behavior outcomes.

Despite the lack of significant interactions, Driving Experience (*X*) consistently predicted key driving behaviors. **In support of H2.2**, Driving Experience negatively predicted acceleration (log ACC_95_ : β = −0.019, *t* = −3.942, *p* < 0.001) and positively predicted steering variability (log Steering_std_ : β = 0.017, *t* = 4.243, *p* < 0.001). However, its effect on speed variability was not significant (log Speed_std_ : β = −0.010, *t* = −1.784, *p* = 0.076).

Extraversion (*M*) had a significant positive effect on acceleration (log ACC_95_ : β = 0.011, *t* = 2.676, *p* < 0.01) and speed variability (log Speed_std_ : β = 0.011, *t* = 2.463, *p* < 0.05), supporting its association with greater fluctuations in driving behavior, as hypothesized in **H2.3**. These findings align with the conclusions of the previous sections, further highlighting Extraversion's role in driving patterns characterized by higher variability in acceleration and speed. Also, consistent with earlier analyses, Extraversion did not significantly affect steering variability (log Steering_std_ : β = 0.004, *t* = 1.159, *p* = 0.248).

Gender, as a covariate, had a significant effect on acceleration (log ACC_95_ : β = −0.076, *t* = −2.009, *p* < 0.05), with male drivers (coded as 1) exhibiting less stable acceleration patterns compared to female drivers (coded as 2). However, Gender did not significantly predict speed variability (log Speed_std_ : β = −0.022, *t* = −0.667, *p* = 0.505) or steering variability (log Steering_std_ : β = −0.003, *t* = −0.108, *p* = 0.914).

These results suggest that while Driving Experience and Extraversion independently influence certain driving behaviors, their interaction effects and the moderating role of arousal states are not significant. The detailed regression results are presented in Table 4.

**Table 4:** Moderated moderation analysis when Extraversion as moderator.

	**Log ACC95 (Y1)**		**Log Speed SD (Y2)**		**Log steering SD (Y3)**	
**Predictors**	**β**	***t***	**β**	***t***	**β**	***t***
Driving experience (X)	–0.019^***^	–3.942	–0.010	–1.784	0.017^***^	4.243
Gender	–0.076^*^	–2.009	–0.022	–0.667	-0.003	–0.108
Extraversion (M)	0.011^***^	2.676	0.011^*^	2.463	0.004	1.159
Arousal states (Z)	–0.011	–0.341	–0.034	–0.976	0.004	0.137
X * M	0.001	0.359	0.002	1.21	–0.000	–0.006
X * Z	0.001	0.047	0.007	0.637	–0.003	–0.335
M * Z	0.011	1.350	0.007	0.740	–0.005	–0.788
X * M * Z	–0.0001	–0.035	–0.002	–0.749	0.0001	0.048
*R* ^2^	0.21	-	0.123	-	0.261	-
*F*	11.138	-	2.084	-	4.950	-

M, Mediator; Z, Moderator in moderation; Y1, output 1; Y2, output 2; Y3, output 3; Significance level:

* *p* < 0.05;

*** *p* < 0.001.

### 5.4 Moderation analysis with neuroticism

The statistic results of Hayes's PROCESS macro (model 3) were shown in Table 5. Since the moderation effect of Neuroticism was observed. The interaction term between Driving Experience and Neuroticism (X*M) significantly predicted acceleration (log ACC_95_ : β = 0.002, *t* = 2.762, *p* < 0.01), speed variability (log Speed_std_ : β = 0.002, *t* = 2.491, *p* < 0.05).

**Table 5:** Moderated moderation analysis when Neuroticism as moderator.

	**Log ACC95 (Y1)**		**Log Speed SD (Y2)**		**Log Steering SD (Y3)**	
**Predictors**	**β**	***t***	**β**	***t***	**β**	***t***
Driving experience (X)	–0.020^***^	–5.5273	–0.008^*^	–1.978	0.0165^***^	5.086
Gender	–0.091^*^	–2.496	–0.041	–1.510	–0.015	–0.456
Neuroticism (M)	–0.001	–0.586	0.006^**^	2.767	0.002	0.619
Arousal States (Z)	–0.011	–0.367	–0.038	–1.464	0.007	0.257
X * M	0.002^**^	2.762	0.002^*^	2.491	0.001	0.856
X * Z	–0.001	–0.162	0.006	0.745	–0.004	–0.589
M * Z	–0.006	–1.311	0.0004	0.104	–0.007	–1.211
X * M * Z	0.0009	0.697	–0.002	-1.171	–0.001	–0.378
*R* ^2^	0.214	-	0.128	-	0.268	-
*F*	12.785	-	2.711	-	4.570	-

M, Mediator; Z, Moderator in moderation; Y1, output 1; Y2, output 2; Y3, output 3; Significance level:

* *p* < 0.05;

** *p* < 0.01;

*** *p* < 0.001.

Then, we reverted our analysis model to a moderation analysis of Neuroticism without the arousal states as variables. We used PROCESS model 1 to continue the test. The results of this analysis can be found in Table 6.

**Table 6:** Moderation analysis when Neuroticism as moderator.

	**Log ACC95 (Y1)**		**Log Speed SD (Y2)**		**Log Steering SD (Y3)**	
**Predictors**	**β**	***t***	**β**	***t***	**β**	***t***
Driving experience (X)	–0.019^***^	–7.596	–0.009^***^	–3.891	0.016^***^	7.080
Gender	–0.100^**^	–2.760	–0.041	–1.325	–0.022	–0.708
Neuroticism (M)	–0.001	–0.398	0.006^**^	2.651	0.002	0.901
X * M	0.002^**^	3.436	0.002^***^	3.870	0.000	0.951
*R* ^2^	0.205	-	0.317	-	0.506	-
Δ*R*^2^ (with X * M)	0.040	-	0.0573	-	0.003	-
*F*	11.809	-	14.973	-	0.904	-

M, Mediator; Y1, output 1; Y2, output 2; Y3, output 3; Significance level:

** *p* < 0.01;

*** *p* < 0.001.

In support of **H2.2**, driving experience negatively predicted acceleration (log ACC_95_ : β = −0.019, *t* = −7.596, *p* < 0.001) and speed variability (log Speed_std_ : β = −0.009, *t* = −3.891, *p* < 0.001). This indicates that experienced drivers exhibit greater stability in both acceleration and speed. Additionally, driving experience positively predicted steering variability (log Steering_std_ : β = 0.016, *t* = 7.080, *p* < 0.001), more experienced drivers exhibit greater fluctuations in steering behavior, which may reflect increased responsiveness or instability depending on the driving context. Collectively, driving experience explained a substantial portion of variance across these behaviors, with *R*^2^ = 0.205, *R*^2^ = 0.317, and *R*^2^ = 0.506 for acceleration, speed, and steering variability, respectively.

The results also supported the moderation role of Neuroticism (**H3.4**) in the relationships between driving experience and both acceleration and speed variability. Neuroticism moderated the effect of driving experience on acceleration [$\Delta R^{2}=0.040,F_{\left(1,\;235\right)}=11.809,p<0.001$] and speed variability [$\Delta R^{2}=0.057,F_{\left(1,\;235\right)}=14.973,p<0.001$]. For steering variability, the interaction term was not significant [$\Delta R^{2}=0.003,F_{\left(1,\;235\right)}=0.904,p=0.342$], suggesting that Neuroticism had a limited moderating role in this domain.

From the bootstrapping results presented in Table 7, the moderation effect of Neuroticism revealed nuanced patterns. For acceleration (log ACC_95_), the effect of driving experience was strongest under low levels of Neuroticism (β = −0.029, 95% CI = [−0.0383, −0.0204]), decreased at mean Neuroticism levels (β = −0.019, 95% CI = [−0.0243, −0.0143]), and further diminished under high Neuroticism (β = −0.009, 95% CI = [−0.0153, −0.0033]). A similar trend was observed for speed variability (log Speed_std_), where the effect was significant under low (β = −0.018, 95% CI = [−0.0260, −0.0105]) and mean levels of Neuroticism (β = −0.009, 95% CI = [−0.0128, −0.0042]), but not significant under high Neuroticism (β = 0.001, 95% CI = [−0.0040, 0.0063]).

**Table 7:** Effect of driving experience on driving behaviors with Conditional Neuroticism.

	**Log ACC95 (Y1)**		**Log Speed SD (Y2)**	
**Neuroticism Condition**	**Effect**	**Boot 95 % CI**	**Effect**	**Boot 95 % CI**
Low (Mean-SD)	**–0.0294**	(–0.0383, –0.0204)	**–0.0182**	(–0.0260, –0.0105)
Mean	**–0.0193**	(–0.0243, –0.0143)	**–0.0085**	(–0.0128, –0.0042)
High (Mean+SD)	**–0.0093**	(–0.0153, –0.0033)	0.0012	(–0.0040, 0.0063)

Boot, Bootstrapping; CI, Confidence Interval. Bold values indicate a significant effect.

Gender, included as a covariate, had a significant negative effect on acceleration (log ACC_95_ : β = −0.100, *t* = −2.760, *p* < 0.01), indicating that male drivers (coded as 1) exhibited less stable acceleration patterns compared to female drivers (coded as 2). However, gender did not significantly predict speed variability (log Speed_std_ : β = −0.041, *t* = −1.325, *p* = 0.186) or steering variability (log Steering_std_ : β = −0.022, *t* = −0.708, *p* = 0.479). Theses results align with the previous analysis.

**Figure 11:**
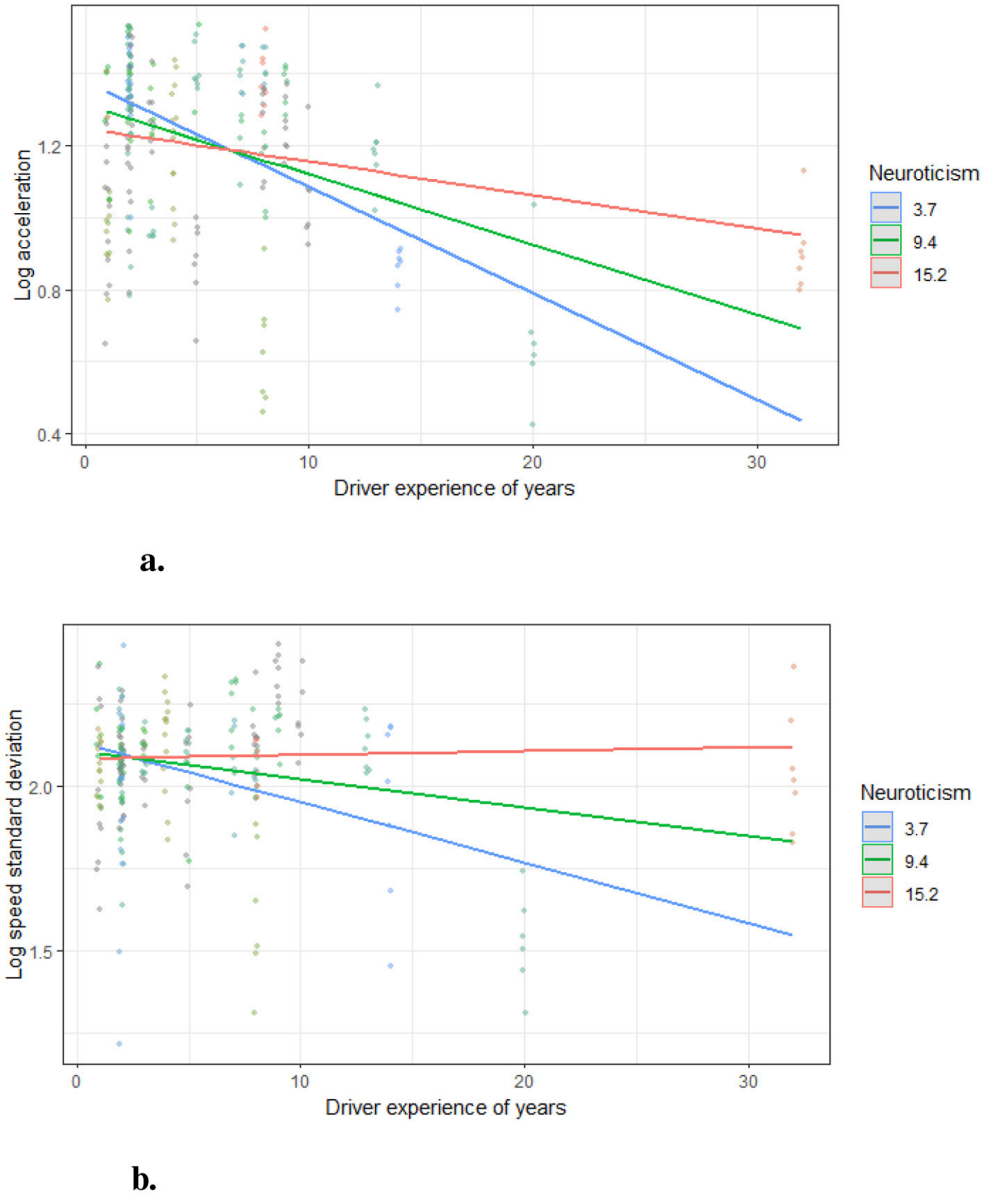
Moderation effect of neuroticism. The Neuroticism levels were grouped by the mean value and +/−1 standard deviation. **(A)** Moderated effect on acceleration. **(B)** Moderated effect on speed standard deviation.

**Figure 12:**
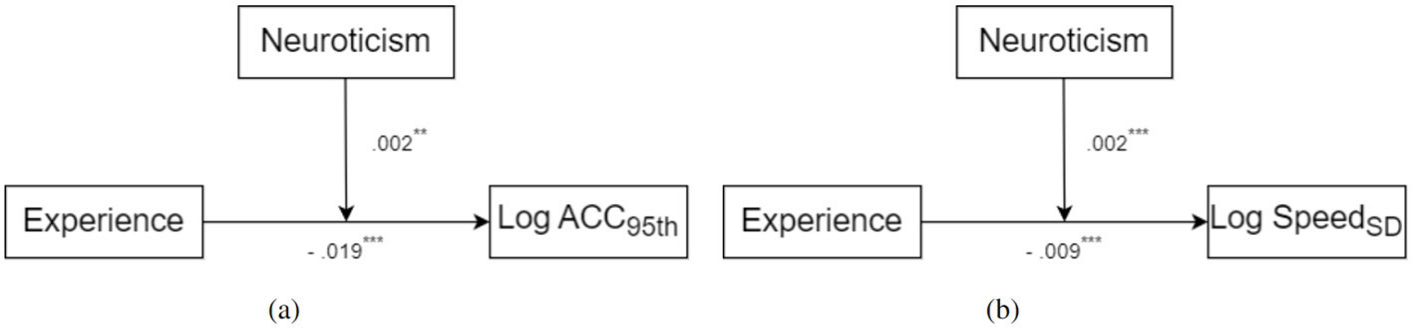
The moderation model. Statistically significant relationships are retained by solid lines. The coefficients are shown next to each regression path. ^**^ indicates *p* < 0.01, ^***^ indicates *p* < 0.001. **(A)** Model 10. **(B)** Model 11.

These findings are visualized in Figure 11, which highlights the moderation effects of Neuroticism on driving behaviors, with a detailed scheme provided in Figure 12. The regression results, presented in Table 6, indicate that driving experience negatively predicted both acceleration and speed variability, suggesting that greater experience contributes to more stable driving behaviors. Neuroticism, however, significantly moderated these relationships, amplifying the effects of driving experience on both acceleration and speed stability, as shown in the bootstrapped results summarized in Table 7. Notably, drivers with lower levels of Neuroticism exhibited more pronounced improvements in stability with increased driving experience, while those with higher Neuroticism showed less stable patterns. However, Neuroticism's moderating influence on steering variability was minimal, reflecting its limited role in shaping this aspect of driving behavior. These results underscore the nuanced interplay between personality traits and experience in shaping driving performance.

### 5.5 Visualization and interpretation of interaction effects

Figures 11, 12 illustrate the interaction effects explored in this study. Below, we provide detailed interpretations of the visualized trends and their alignment with the study's hypotheses.

#### 5.5.1 Moderated mediation effect of extraversion

Figures 9A, B present the moderated mediation effect of Extraversion under different Arousal States (low and high). In low-arousal conditions, acceleration decreases as age increases, with Extraversion levels minimally affecting the slope of the decline (Figure 9A). However, in high-arousal conditions, the lines diverge more prominently, suggesting that higher Extraversion levels amplify the age-related decline in acceleration. These findings support **H4**.

Similarly, Figure 9B shows that in high-arousal conditions, Extraversion significantly influences the relationship between Age and Speed Variability. The steepest decline is observed for individuals with high Extraversion, emphasizing the interactive role of personality traits and situational factors in shaping driving behaviors.

#### 5.5.2 Moderation effect of Neuroticism

Figure 11A demonstrates the moderation effect of Neuroticism on the relationship between Driving Experience and acceleration (log ACC_95_). The x-axis represents the years of driving experience, while the y-axis shows the log-transformed acceleration. The lines correspond to different levels of Neuroticism: low (blue), mean (green), and high (red). It is evident that as driving experience increases, acceleration decreases across all Neuroticism levels. However, the slope of the decrease is steepest for drivers with low Neuroticism, suggesting a stronger negative relationship between driving experience and acceleration in this group.

In contrast, Figure 11B illustrates the moderation effect on Speed Variability (log Speed_std_). Similar trends are observed, with speed variability decreasing as driving experience increases. Drivers with low Neuroticism exhibit the steepest decline, indicating that higher Neuroticism levels attenuate the relationship between driving experience and speed variability. These results align with **H3.4**, highlighting Neuroticism as a significant moderator.

These visualizations provide critical insights into the complex interplay between personality traits, situational factors, and driving behaviors, reinforcing the theoretical framework supporting this study.

### 5.6 Analysis summary

To summarize our analysis, we established several models to find the interactions between age, driving experience, personality, and driving behaviors under different arousal states. The statistical results suggested that our Hypothesis 1 was partly supported that the age of drivers was positively associated with Extraversion (H1.1 was supported), however, the relation between age and Neuroticism was not found (H1.2 not supported). As for Hypothesis 2, H2.1, H2.2, H2.3, and H2.4 were partly supported. Driver's age and driving experience predicted lower acceleration (lower log*ACC*_95_), higher speed stability (lower log*Speed*_*std*_), but larger steering instability (higher log*Steering*_*std*_). Additionally, Extraversion was a positive predictor of higher acceleration (higher log*ACC*_95_) and higher speed instability (higher log*Speed*_*std*_). Neuroticism was found to be positively associated with higher speed instability (higher log*Speed*_*std*_). Moreover, The mediation role of Extraversion (H3.1 was supported) and the moderation role of Neuroticism were found (H3.4 was supported). At last, from H4, we found significant evidence that the arousal states moderated the mediation effect of Extraversion on the relation between age and driving behaviors (log*ACC*_95_ and log*Speed*_*std*_). In this way, H4 was suggested and Extraversion was sensitive to the variation of arousal states.

## 6 Discussion

The current study aimed to explore the relationships between personality traits, emotional states, driving experience, and age in shaping driving behaviors among Chinese drivers. By employing moderated mediation and moderation models, this study provides novel insights into how individual differences and situational factors interact to influence driving performance.

### 6.1 Age and personality

Our findings supported **H1.1**, demonstrating that age positively predicts Extraversion. This aligns with previous research suggesting that Extraversion, particularly its social dominance component, increases with age due to life experiences fostering independence, self-confidence, and assertiveness (Roberts et al., 2006; Ashton and Lee, 2016). These findings are consistent with the idea that older individuals exhibit greater social confidence and reduced social anxiety, as highlighted by Bleidorn et al. (2019).

However, **H1.2** was not supported, as no significant relationship was observed between age and Neuroticism. This discrepancy with prior research, which often shows a decline in Neuroticism with age (Roberts et al., 2006; Stieger et al., 2021), may reflect uneven age distribution in our sample or cultural differences influencing emotional stability (Yik et al., 2002; Kuppens et al., 2017). These results suggest that further research is needed to explore this relationship using more balanced samples and culturally sensitive measures.

### 6.2 Age, driving experience, and personality

Age and driving experience emerged as pivotal factors influencing driving behaviors, reflecting distinct yet interconnected effects. Supporting **H2.1**, higher age was associated with reduced acceleration variability and improved speed stability, aligning with previous research that highlights older drivers' better emotional regulation and risk assessment skills (Harré et al., 2000; Taubman-Ben-Ari and Skvirsky, 2016). However, increased steering variability with age suggests adaptive adjustments rather than instability. Older drivers may employ more nuanced steering strategies to manage complex driving scenarios effectively, consistent with findings by Pawar and Velaga (2021) and Macdonald and Hoffmann (1980). These results reinforce the view that age contributes to safer driving behaviors but also indicates the complexity of its influence on different driving dimensions. While age significantly influences personality traits, particularly Extraversion, its role in shaping driving behaviors highlights both direct and indirect pathways through emotional and behavioral adaptations.

Supporting **H2.2**, greater driving experience was associated with reduced acceleration variability and enhanced speed stability, reflecting the benefits of experience in fostering smoother and more controlled driving behaviors (Crundall et al., 1999; Evans et al., 2022). Experienced drivers' ability to anticipate hazards and maintain consistent driving patterns underscores their superior adaptability in diverse traffic situations. However, the observed increase in steering variability with experience, similar to the effects of age, suggests that these adjustments may reflect deliberate and adaptive strategies rather than instability.

Extraversion and Neuroticism further modulated these effects, providing insights into how personality traits influence driving behaviors. Supporting **H2.3**, Extraversion was associated with higher acceleration and poorer speed stability, consistent with prior research linking Extraversion to stimulation-seeking and risk-prone behaviors (Eysenck and Eysenck, 1975). Drivers with high Extraversion levels may engage in behaviors that compromise stability, particularly in dynamic driving conditions. Conversely, **H2.4** was partially supported: Neuroticism was associated with higher acceleration variability, suggesting a link between emotional instability and less controlled driving behaviors. However, its impact on speed and steering stability was less pronounced, highlighting the nuanced role of Neuroticism in shaping driving patterns directly.

In summary, the interplay between age, driving experience, and personality traits shapes driving behaviors in multifaceted ways. While age and experience generally promote safer driving styles, the influence of Extraversion and Neuroticism underscores the importance of considering individual differences in personality when evaluating driving performance. These findings contribute to a more nuanced understanding of the factors underpinning driving stability and risk, emphasizing the need for personalized approaches in driver training and interventions.

### 6.3 Personality traits and emotional modulation

Extraversion played a significant mediating role in driving behaviors, supporting **H3.1**. The findings revealed that age indirectly influenced driving behaviors through Extraversion. Older drivers tended to exhibit higher levels of Extraversion, which in turn increased their acceleration and speed variability. This result aligns with Eysenck and Eysenck (1975)'s theory, suggesting that Extraverts are more stimulation-seeking and prone to risk-taking behaviors. Moreover, the moderated mediation effect highlights the interplay between Extraversion and emotional regulation (supporting **H4**), particularly under high-arousal conditions. In such states, the effects of Extraversion on driving behaviors were amplified, leading to heightened acceleration and speed instability. These findings are consistent with Brummer et al. (2014), who emphasized the significant role of Extraversion in shaping emotion regulation strategies.

The mediation pathway from age to driving behaviors via Extraversion underscores the complex influence of personality maturation on driving outcomes. Older drivers, characterized by higher social dominance and stimulation-seeking tendencies, may exhibit driving behaviors that reflect both cautious decision-making and occasional risk-taking. This duality aligns with the findings of Roberts et al. (2006), who noted an increase in Extraversion with age, particularly in traits like social dominance and self-confidence. Thus, while age positively contributes to personality development, the behavioral outcomes mediated through Extraversion may sometimes counteract the stabilizing effects of age on driving.

However, **H3.3** was not supported, as the interaction between Extraversion and driving experience did not significantly influence driving behaviors. This suggests that the stimulation-seeking tendencies associated with Extraversion may not directly interact with the learning and adaptive processes facilitated by driving experience. According to Zuckerman (1995, 1991), structured environments such as driving may impose external constraints that limit the expression of Extraversion-related tendencies.

Conversely, Neuroticism did not significantly mediate the relationship between age and driving behaviors, contradicting **H3.2**. Although Neuroticism is typically linked to emotional instability and heightened stress sensitivity (Eysenck and Eysenck, 1975), its indirect influence was not evident in our data. This discrepancy may reflect cultural or methodological factors, as suggested by Stieger et al. (2021), who emphasized the role of context in shaping Neuroticism-related outcomes.

In contrast, **H3.4** was supported, as Neuroticism moderated the relationship between driving experience and driving behaviors. Specifically, Neuroticism attenuated the positive effects of driving experience on acceleration and speed stability. Drivers with lower levels of Neuroticism benefited more from increased driving experience, exhibiting greater improvements in stability. Conversely, those with higher Neuroticism showed diminished benefits, reflecting the disruptive influence of emotional instability on learning and adaptation. These results align with Castillo-Gualda et al. (2019), who noted that Neuroticism is associated with emotional exhaustion, potentially hindering drivers' ability to effectively leverage their experience.

Together, these findings illustrate the dual influence of age and personality traits on driving behaviors. While age promotes the development of Extraversion, leading to riskier tendencies under high-arousal conditions, Neuroticism disrupts the stabilizing effects of driving experience. These insights underscore the importance of considering both age-related personality maturation and the dynamic role of emotional traits in shaping driving performance.

### 6.4 Integrating cultural and contextual factors

This study's focus on Chinese drivers offers valuable insights into how cultural contexts may influence the relationships among personality traits, emotional states, and driving behaviors. While our findings regarding Extraversion are consistent with global research, they also suggest potential cultural influences that require further investigation. For instance, comparative studies of drivers in Japan, China, and Vietnam have revealed differences in aberrant driving behaviors, with Vietnamese drivers exhibiting higher levels of such behaviors and Japanese drivers displaying fewer aggressive violations. These findings underline how cultural norms and societal values can shape driving behaviors in distinct ways (Hussain et al., 2019). Similarly, research comparing Chinese and Pakistani drivers has highlighted differences in dangerous and positive driving styles, emphasizing the importance of cultural contexts in understanding driving patterns (Yousaf and Wu, 2024).

Our observations of cautious driving tendencies among female drivers, though limited by sample size, align with broader studies linking gender norms and stereotypes to driving behaviors. For example, research has shown that adherence to male stereotypes is associated with increased risk-taking, whereas female drivers are often characterized as more cautious (Granié, 2024; Castro-Nuno and Lopez-Valpuesta, 2023). Although our findings cannot provide definitive conclusions, they highlight potential cultural and gender-related factors that merit further exploration.

Future research should adopt a cross-cultural approach to examine these dynamics in greater detail. Investigating cultural dimensions, such as individualism vs. collectivism, could shed light on how personality traits like Extraversion interact with cultural contexts to influence driving behaviors. Additionally, designing culturally specific interventions based on these insights could help enhance road safety while respecting local norms and values. Such efforts could bridge the gap between theoretical research and practical applications, contributing to safer driving practices globally.

### 6.5 Implications for driving safety and emotional regulation

Our findings offer valuable practical implications for improving traffic safety and advancing our understanding of the interplay between personality traits, emotional regulation, and driving behaviors. The observed moderated mediation effects underscore the importance of tailoring training programs to address individual differences in personality and emotional responses. Specifically, Extraverted drivers, who exhibit heightened risky behaviors under high-arousal conditions, would benefit from targeted interventions focusing on emotional regulation. Similarly, addressing Neuroticism in driving interventions could amplify the stabilizing effects of driving experience for drivers with high emotional instability.

From a theoretical perspective, these findings challenge traditional assumptions about emotional instability and driving behavior. While Neuroticism is typically associated with emotional instability, its limited role in our study suggests that cultural or contextual factors may shape the expression of personality traits in driving contexts. Conversely, the pronounced influence of Extraversion highlights the dynamic interaction between personality and situational factors, offering new insights into the complexity of driving behavior.

To enhance road safety, we propose two specific recommendations aimed at applying our research findings effectively:

1. **Enhancing driver training:** Traditional driver training programs focus primarily on basic skills and managing dangerous situations in typical scenarios. However, many road accidents occur when drivers experience emotional arousal, such as anger or stress. To address this gap, we recommend incorporating psychological assessments and emotional regulation strategies into training programs. Specifically:
  - **Identify emotionally susceptible drivers:** Use psychological assessments to identify drivers who are highly susceptible to emotional arousal, particularly those with high levels of Extraversion or Neuroticism.
  - **Simulate emotional arousal scenarios:** Employ virtual reality (VR) or augmented reality (AR) technology to create realistic high-stress scenarios during training. This allows drivers to practice managing their emotions in controlled environments.
  - **Emotional competency tests:** Include emotional competency evaluations as part of the training process. Drivers should demonstrate their ability to navigate emotionally charged situations effectively before completing the program.
2. **Advanced driving assistance systems:** Current smart car technologies often rely on facial emotion recognition or fatigue detection to identify potential risks, but these methods may react too late to prevent accidents. We propose developing advanced driving assistance systems (ADAS) that integrate personality, age, and gender data to provide personalized support. Specifically:
  - **Personalized emotional support:** Implement features offering regular emotional counseling for drivers with high Extraversion or Neuroticism to help them manage their emotions effectively while driving.
  - **Active human-computer interactions:** Design active human-computer interaction (HCI) systems for younger drivers to reduce human errors. Real-time feedback and guidance tailored to the driver's emotional state and behavior can improve safety.
  - **Proactive safety features:** Integrate proactive safety measures that detect and respond to emotional states or abnormal driving behaviors before they escalate. For instance, calming prompts or adjustments to driving settings can help reduce stress in real-time.

By implementing these targeted recommendations, driver training programs can better prepare individuals to handle emotional challenges on the road, while advanced ADAS can enhance the safety features of next-generation vehicles. Together, these strategies offer a comprehensive approach to mitigating risky driving behaviors and fostering safer road environments.

## 7 Conclusion and limitation

To summarize, this study provides insights into the relationship between demographic factors, personality traits, and driving behaviors under varying arousal conditions. Two moderated mediation models and two moderation models were developed to explain acceleration variability and speed stability, with age, driving experience, Extraversion, and Neuroticism as the main predictors. Our findings support the significant impact of age and driving experience on driving behaviors, with Extraversion playing an indirect mediating role and arousal states acting as a moderator. Additionally, Neuroticism moderated the relationship between driving experience and driving behaviors. These findings highlight the negative impact of Extraversion and Neuroticism on driving performance, suggesting that they are potential risk factors for unsafe driving. Particularly, the interaction between Extraversion and emotional arousal was shown to increase driving risks. This study contributes to the literature on driving behaviors and individual differences, offering valuable insights for traffic safety education and targeted accident prevention measures. Based on these findings, we emphasize the need to consider driver demographics and personality traits as fundamental factors underlying emotional driving. Further research is encouraged to explore the complex interactions among these factors to better understand driving styles and drivers' strategies for emotional regulation.

This study also has several limitations. Firstly, the sample size is relatively small, with only 40 drivers and 240 driving records collected, making it difficult to generalize the findings to the wider population of Chinese drivers. Additionally, potential bias in sample demographics, such as the predominance of male participants and limited age diversity, may further constrain the generalizability of our findings. These limitations should be acknowledged when interpreting the results. Also, the age distribution in our sample, with a significant proportion of participants under 25 years old, may limit the generalizability of the findings to the broader driving population. Secondly, while this study examined the impact of arousal states (i.e., high and low) on driving behaviors, it did not account for the full spectrum of arousal levels. The binary classification of arousal may oversimplify its effects, as individual differences in arousal intensity could play a significant role in shaping driving behaviors. Future studies should adopt more granular measures of arousal to capture nuanced effects.

Thirdly, this study provides only a limited view of individual differences and their relationship to driving behaviors. Individual differences encompass a wide range of factors, such as cultural and health distinctions, which were not addressed in this paper. Furthermore, some key aspects of driving behavior, including eye movements and body posture, remain unexplored. Moreover, gender differences, although identified as significant in our findings, were not deeply analyzed due to sample imbalances. This omission limits our understanding of gender-specific driving behaviors, highlighting the need for balanced sampling and targeted analyses in future studies. Additionally, there is room for improvement in the measurement of steering performance, as the observed steering variability may reflect both adaptive strategies and potential instability.

The laboratory setting of this study represents another limitation. While the fixed-base driving simulator allowed for controlled experimentation, it lacks motion and sensory feedback, which may reduce the ecological validity of the findings. This limitation underscores the importance of complementing simulator-based research with naturalistic driving studies to better capture real-world behaviors. Lastly, our dataset does not conform to a normal distribution, which may affect the reliability and generalizability of our results. While the complexity of data collection and emotional elicitation presents significant challenges, this limitation highlights the need for more robust statistical techniques and experimental designs in future research.

In conclusion, addressing these limitations in future studies could provide a more comprehensive understanding of the factors influencing driving behaviors. Specifically, larger and more diverse samples, more detailed measures of arousal, in-depth analyses of gender effects, and real-world driving data could enhance the reliability and applicability of research findings in this field.

## Data Availability

The original contributions presented in the study are included in the article/supplementary material, further inquiries can be directed to the corresponding author.

## Footnotes

^1^The study includes a total of 40 participants, with one driver aged above 54 years, and males comprising 77.5% of the sample.

^2^The EEG data is not used in our research.
